# Rationale and design of the randomised, controlled Percutaneous coronary intervention using Assisted Robotic TechnologY (PARTY) trial

**DOI:** 10.1136/openhrt-2024-002950

**Published:** 2024-11-21

**Authors:** James Leung, James Xu, John K French, Hashim Kachwalla, Krishna Kaddapu, Tamer Badie, Christian Mussap, Rohan Rajaratnam, Dominic Y Leung, Sidney T Lo, Craig Juergens

**Affiliations:** 1 Department of Cardiology, Liverpool Hospital, Liverpool, New South Wales, Australia; 2 University of New South Wales, Sydney, New South Wales, Australia; 3 Department of Cardiology, Campbelltown Hospital, Campbelltown, New South Wales, Australia

**Keywords:** CORONARY ARTERY DISEASE, Percutaneous Coronary Intervention, Coronary Angiography

## Abstract

**Introduction:**

Percutaneous coronary intervention (PCI) is performed by interventional cardiologists who, over their career, are exposed to substantial X-ray radiation and also face potential musculoskeletal injuries from the prolonged wearing of heavy protective equipment. Patients are also at risk from radiation exposure. Robotic-assisted PCI (R-PCI) allows millimetre control of the coronary guide-catheter, guidewires and intracoronary devices via three joysticks and touchscreens located within in a shielded ‘cockpit’, away from radiation. Initial studies have demonstrated efficacy and safety of R-PCI with significant radiation reductions. However, these studies are non-randomised, registry based or observational data with short clinical follow-up. We aim to perform the first randomised controlled trial investigating R-PCI compared with manual PCI (M-PCI) with longer clinical follow-up.

**Methods and analysis:**

The Percutaneous coronary intervention using Assisted Robotic TechnologY (PARTY) trial is a single-centre, prospective, randomised, open-label clinical trial comparing R-PCI to M-PCI. Eligible patients between 18 and 85 years undergoing coronary angiography, and are subsequently deemed to require PCI, will be screened. A key exclusion criterion is if the operator determines that the participant or coronary anatomy is unsuitable for R-PCI. Patients will be randomised to R-PCI or M-PCI in a 1:1 fashion. The primary outcome is radiation exposure to the patient, as measured by personal radiation dosimeters. Other procedural, safety and efficacy outcomes will be compared. Patients will be followed up until hospital discharge (or 72 hours, whichever occurs first), and then by telephone at 30 and 365 days.

**Trial registration number:**

ACTRN12623000480684.

**WHAT IS ALREADY KNOWN ON THIS TOPIC**

- Robotic-assisted percutaneous coronary intervention (R-PCI) has been shown to be as safe and effective as manual PCI in non-randomised and observational studies, case series and registries. There have been no randomised trials comparing R-PCI to M-PCI. Furthermore, it is unclear if there are other additional benefits apart from reduction in radiation exposure to the operators.

**WHAT THIS STUDY ADDS**

- To our knowledge, the Percutaneous coronary intervention using Assisted Robotic TechnologY trial is the first randomised controlled trial worldwide to evaluate the effectiveness of R-PCI compared with M-PCI with longer clinical follow-up. We will be examining radiation exposure to patients and staff, as well as other potential benefits of R-PCI.

**HOW THIS STUDY MIGHT AFFECT RESEARCH, PRACTICE OR POLICY**

- We hope our research will provide additional robust evidence towards the potential benefits of R-PCI. These results may encourage the increasing uptake of R-PCI worldwide.

## Introduction

 Percutaneous coronary intervention (PCI) has established itself as one of the core treatments for coronary artery disease, especially in acute coronary syndromes.^1^ Since first performed in 1977, there have been significant improvements in this procedure. Improved coronary stent design, superior stenting techniques and the development of intracoronary physiology and intracoronary imaging have improved cardiovascular outcomes for patients undergoing PCI.^1^ However, the requirement for X-ray radiation exposure to operator and patient during the procedure persists. Over the course of an interventional cardiologist’s career, cumulative radiation exposure may cause significant morbidity and mortality, particularly cataracts^2^ and brain tumours.^3^ Interventional cardiologists also have increased orthopaedic injury rates from sustained wearing of heavy lead protection equipment.^4 5^ Patients are also at risk of radiation-induced harms, including deterministic (eg, skin erythema and ulceration) and stochastic effects (eg, malignancy).^6^

**Figure 1:**
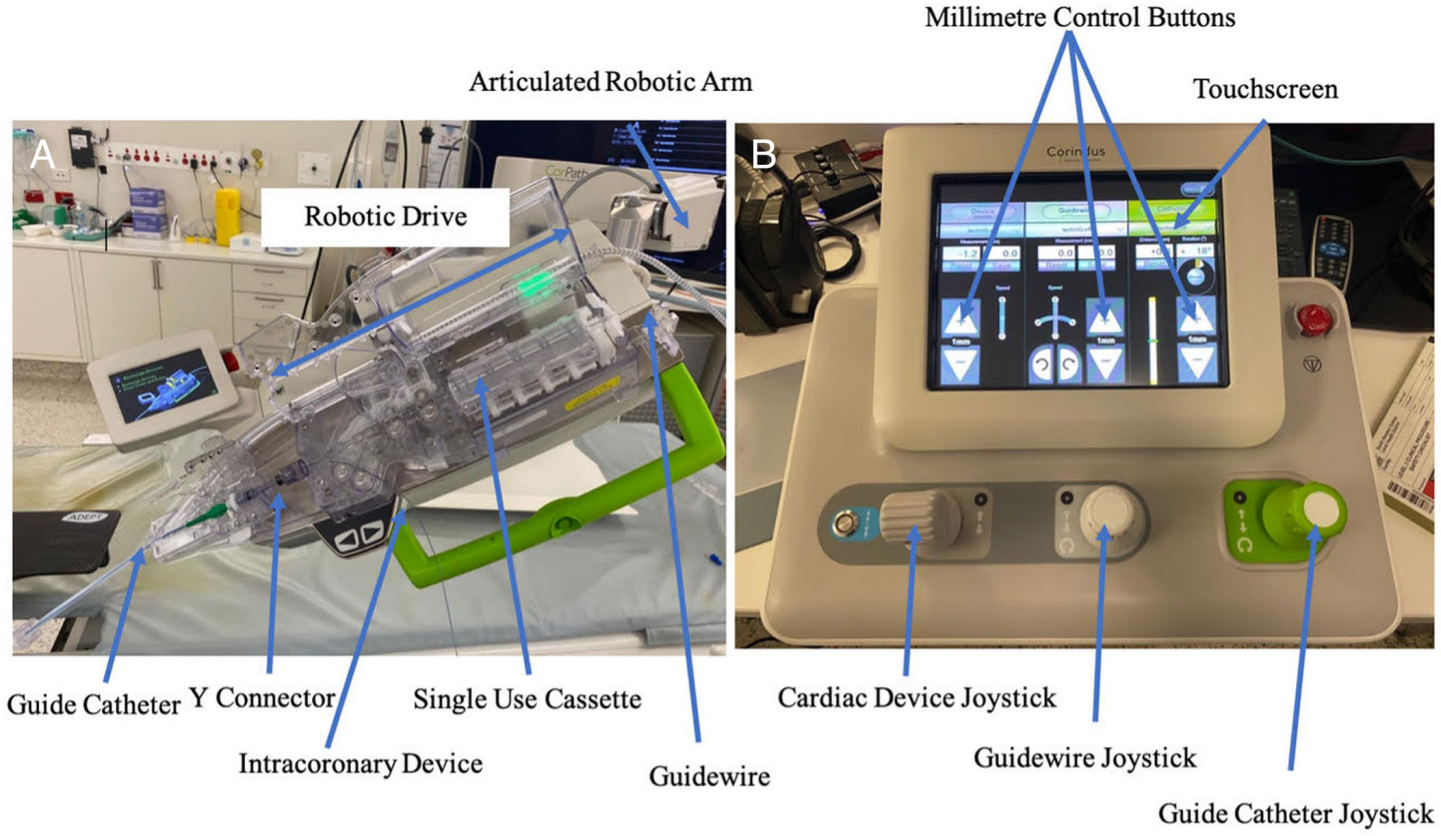
Robotic setup including (A) bedside robotic drive and (B) cockpit console with joysticks and touchscreen.

Robotic-assisted PCI (R-PCI) has been developed to allow the interventional cardiologist millimetre control of the coronary guiding catheter, intracoronary wires and devices. Currently, the two major companies in the R-PCI field include Robocath (Rouen, France) and Corindus Vascular Robotics (Massachusetts, USA). The setup comprises a bedside robotic drive and shielded ‘cockpit’. The bedside robotic drive consists of a robotic cassette into which the guiding catheter, guidewires and intracoronary devices are loaded (figure 1A). The cockpit includes three joysticks allowing independent control of the guiding catheter, coronary wire and intracoronary device (figure 1B). This cockpit is situated outside the catheter laboratory, away from the radiation. Despite robotic assistance, manual inputs are still required. Arterial access and the diagnostic angiogram are still performed manually. Furthermore, the operator must be available to ‘bail out’ the robot and take over should it be unable to complete the PCI.

Initial clinical evidence for R-PCI has been encouraging for safety and efficacy. The Percutaneous Robotically Enhanced Coronary Intervention study was the first non-randomised clinical registry investigating R-PCI.^7^ Using the first-generation CorPath 200 (Corindus Vascular Robotics, Massachusetts, USA) in 164 patients across nine sites, clinical success, defined as<30% residual stenosis at completion of PCI with Thrombolysis In Myocardial Infarction 3 flow without major adverse cardiovascular events (MACE), was achieved in 160 patients (97.6%). Procedural success, defined as successful completion of the PCI procedure without conversion to manual operation, occurred in 162 patients (98.8%). Apart from four periprocedural non-Q-wave myocardial infarctions, there were no major cardiovascular complications at 30 days (eg, deaths, Q-wave myocardial infarctions or repeat revascularisation). Notably, the primary operator experienced a 95.2% reduction in radiation exposure while in the cockpit compared with at the bedside. A propensity score-matched analysis of a large R-PCI cohort identified significant radiation reductions of almost 20% to patients undergoing R-PCI compared with manual PCI (M-PCI).^8^ A recent meta-analysis of seven studies totalling 2230 patients identified significant reductions in operator radiation exposure (mean difference −442.32 mGy, p=0.002), fluoroscopy time (mean difference −1.45 min, p=0.05) and contrast usage (mean difference −18.28 mL, p<0.00001) with R-PCI compared with M-PCI.^9^ Importantly, the rates of procedural success and complications were no different between the groups. However, no randomised clinical trials have been performed worldwide evaluating R-PCI with longer clinical follow-up.

## Methods and analysis

### Study design

**Figure 2:**
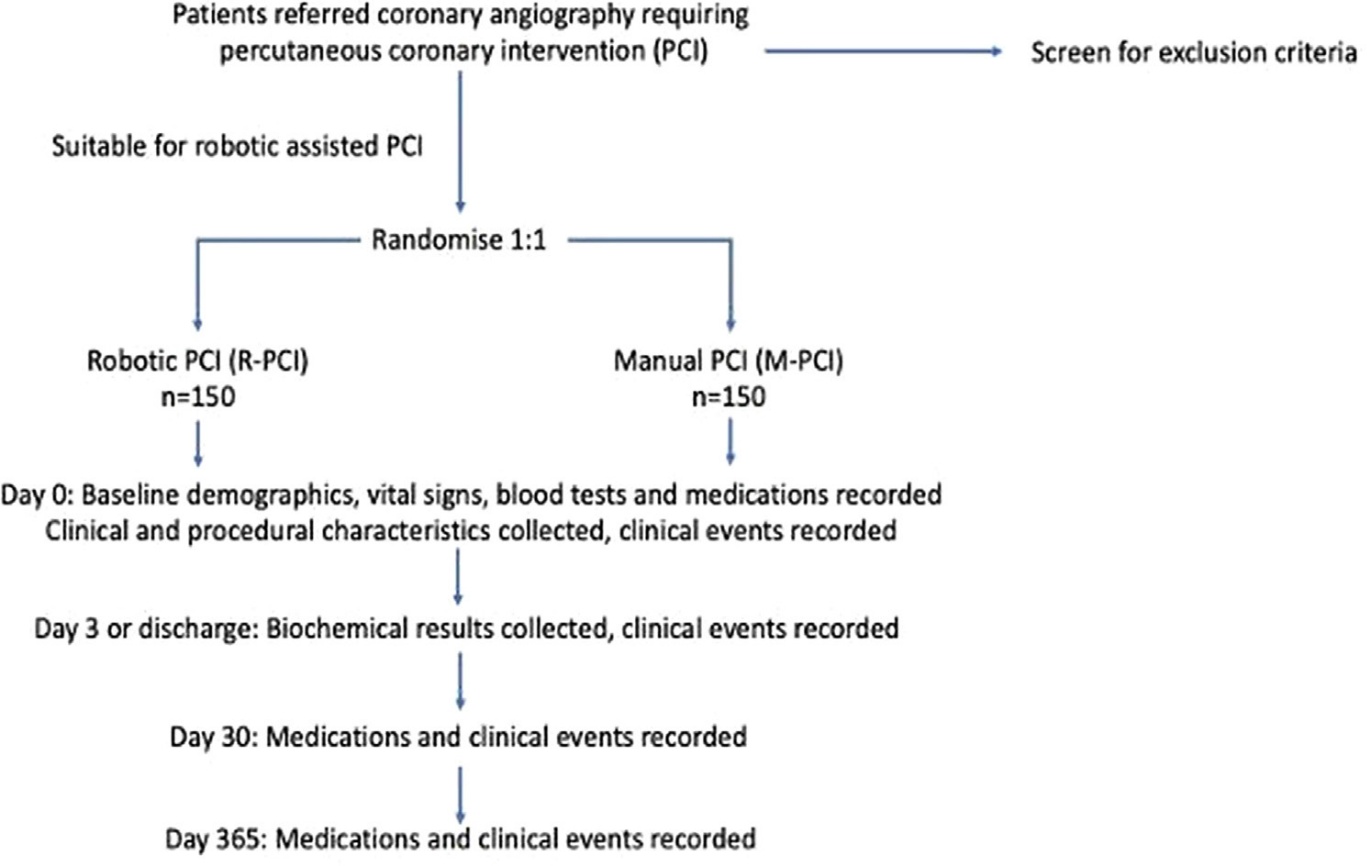
Study flow diagram of the Percutaneous coronary intervention using Assisted Robotic TechnologY trial.

The Percutaneous coronary intervention using Assisted Robotic TechnologY (PARTY) trial is a single-centre, prospective, open label, unblinded randomised controlled trial comparing the safety, efficacy and procedural characteristics of R-PCI using the second generation CorPath GRX system (Corindus Vascular Robotics, Massachusetts, USA), with conventional M-PCI in patients undergoing PCI (figure 2).

The PARTY trial will aim to recruit 300 patients in a 1:1 fashion to R-PCI and M-PCI. Box 1 lists key inclusion and exclusion criteria. All patients aged 18–85 years undergoing coronary angiography at a single tertiary referral hospital who require PCI will be screened for recruitment. Patients presenting with stable coronary artery disease as well as acute coronary syndromes will be eligible for recruitment. Acute ST elevation myocardial infarction patients presenting for primary PCI will be excluded from the trial. A total of three CorPath GRX robots have been installed in the three cardiac catherisation laboratories to maximise patient recruitment. Eligibility will be determined by either the principal investigator or subinvestigator performing the procedure. Patients must provide written informed consent. Key exclusion criteria are if the operator determines that the participant or coronary anatomy is unsuitable for R-PCI. Examples of unsuitable coronary anatomy include chronic total occlusions, bifurcation lesions requiring two-stent strategy, highly tortuous or calcified arteries or complex lesions requiring advanced calcium modification (eg, atherectomy). Intravascular imaging during PCI is encouraged but is left to the discretion of the operator. Once enrolled, patients will be randomised via an electronic web-based randomisation schedule in a 1:1 fashion. Baseline patient demographics, comorbidities and medications will be recorded. Preprocedural pathology results will be collected. Trial participants will be allowed to continue all usual medications during the trial and have no restrictions on further care, treatments or interventions deemed appropriate by their treating clinicians.

Box 1 **Key inclusion and exclusion criteria**

**Inclusion criteria**

1. Age 18–85 years.
2. Participants with coronary artery disease with clinical indication for percutaneous coronary intervention (PCI).
3. Participant deemed appropriate for robotic-assisted PCI (R-PCI).
4. Able to provide a signed and dated informed consent form.

**Exclusion criteria**

1. The investigator determines that the participant or the coronary anatomy is not suitable for R-PCI (eg, chronic total occlusion lesions, bifurcation lesions requiring two-stent strategy, highly tortuous or calcified arteries or complex lesions required advanced calcium modification (eg, atherectomy)).

### Study endpoints

The primary endpoint for the trial is patient radiation exposure, as measured via a personal radiation dosimeter worn by the patient at (or near) the left shoulder. Radiation exposure to the operator, assistant and nurse will be measured as secondary endpoints via personal dosimeters. For proceduralists and assistants, the Polimaster PM1610 X-ray and Gamma Radiation Personal Dosimeter (Minsk, Belarus) and the RaySafe i3 Real-time Radiation Dosimeter (Billdal, Sweden) will be worn outside the lead protection gowns, side by side, in a standardised position in the top pocket on the left side of the chest, over the heart. The patient dosimeter will be positioned at (or near) the left shoulder. Radiation exposure will be measured in micro Sieverts (μSv). Overall, procedural radiation measures such as fluoroscopy time (minutes), air kerma (milliGray) and dose area product (deciGray·metre^2^) will be recorded.

While fluoroscopy settings during PCI will be at the operator’s discretion, they are encouraged to use standardised radiation settings and benchmarks to minimise radiation exposure. These best practices for coronary angiography are well known, established and widely published.^10^ Fluoroscopy will be recommended at 7.5 frames/second and cine acquisition at 10 frames/second. The operator will be encouraged to use as large a field size as possible, to minimise extreme angulation and to incorporate filters and collimation to concentrate on the area of interest while minimising radiation exposure to non-essential regions. The assistant and scrub nurse will be instructed to stand as far away as possible during PCI. As is standard practice, the use of ceiling-mounted upper body shielding and lower body apron shielding mounted underneath the patient table will be mandatory. The catheterisation table will be elevated as much as possible to maximise distance between patient and X-ray source. Furthermore, the distance between the detector and patient will be minimised to reduce radiation exposure from scatter.

R-PCI cases will be performed in a cardiac catheterisation lab using a Siemens (Munich, Germany) X-ray system. Inbuilt into this system are the Combined Applications to Reduce exposure (CARE) features to reduce intraprocedural radiation exposure. Standard features within the cardiac catheterisation laboratory which will be used during all R-PCI cases include:

1. CAREvision (variable fluoroscopy pulse rates).
2. CAREfilter (automatic copper filtration thickness adjustment to reduce patient entrance dose).
3. CAREprofile (radiation free collimation/filter positioning using the last image hold).
4. CAREposition (radiation free positioning of X-ray area of focus).

Secondary endpoints evaluating other performance, safety and procedural outcomes will be collected and are summarised in Box 2. Equipment usage and other procedural data will be recorded to allow a biostatistician to perform a health economics analysis. Key performance measures include clinical success, defined as less than 30% residual stenosis (by visual estimation) post-PCI, without in-hospital MACE and procedural success, defined as successful completion of the PCI without unplanned conversion to manual operation. MACE is defined as either cardiac death, clinically relevant myocardial infarction after coronary revascularisation, or ischaemia-driven target vessel revascularisation. Clinically relevant myocardial infarction after coronary revascularisation will be defined as per the fourth Universal Definition of PCI-related myocardial infarction, which states.^11^

- For participants with normal baseline cardiac biomarkers.
  - Cardiac troponin T (cTNT)>5 times the 99th percentile of upper reference limit (URL).
- For participants with elevated baseline cardiac biomarkers.
  - Rise in post procedure cTNT by >20% and be at least >5 times the 99th percentile of URL and at least one of the following:
  - New ischaemic ECG changes.
  - Development of new pathological Q waves.
  - Imaging evidence of new loss of viable myocardium or new regional wall motion abnormality in a pattern consistent with an ischaemic aetiology.
  - Angiographic findings consistent with a procedural flow limiting complication such as coronary dissection, occlusion of a major epicardial artery or a side branch occlusion/thrombus, disruption of collateral flow or distal embolisation.

Box 2 **Primary and secondary endpoints**

**Overall study aim:**

1. To assess the safety, effectiveness including the clinical and technical performance and cost analysis of robotic-assisted percutaneous coronary intervention (PCI) versus manual PCI.

**Primary endpoint:**

1. Radiation exposure to the patient.

**Secondary endpoints:**

**Performance measures:**

1. Clinical success: less than 30% residual stenosis (visual estimate) post PCI, without in-hospital major adverse cardiovascular events.
2. Procedural success: successful completion of the PCI without unplanned conversion to manual operation.

**Safety measures:**

1. Radiation exposure to operator, assistant and nurse.
2. Major adverse cardiovascular events.
3. All serious adverse events.

**Procedural characteristics:**

1. Total procedure time.
2. Total PCI time.
3. Fluoroscopy time.
4. Robot set up time.
5. Contrast usage.
6. Equipment utilisation.
7. Robotic/manual lesion and stent length comparison.
8. Operator and staff workload survey.

Safety outcomes including MACE and other serious adverse events (eg, bleeding, vascular damage, contrast induced nephropathy) will be recorded. These safety outcomes will be adjudicated by a blinded and independent data safety and monitoring adjudication board of interventional and non-interventional cardiologists (see safety monitoring section). Bleeding complications will be adjudicated using the Bleeding Academic Research Consortium classification. Other procedural characteristics to be collected are included in Box 2.

Patients are allowed to withdraw from the trial at any time. In this case, their data collected up to their withdrawal will be included in the analysis. These patients will continue to have their medical treatments as determined by their usual clinicians.

### Other trial measures

There are additional potential benefits of R-PCI on top of radiation reduction benefits to the operator and patient^9^ that will be investigated. These include potentially reduced operator strain and more precise lesion and stent assessment. These factors will be assessed via a quantitative survey of overall workload and a robotic versus manual lesion and stent length comparison.

### Workload assessment survey

**Figure 3:**
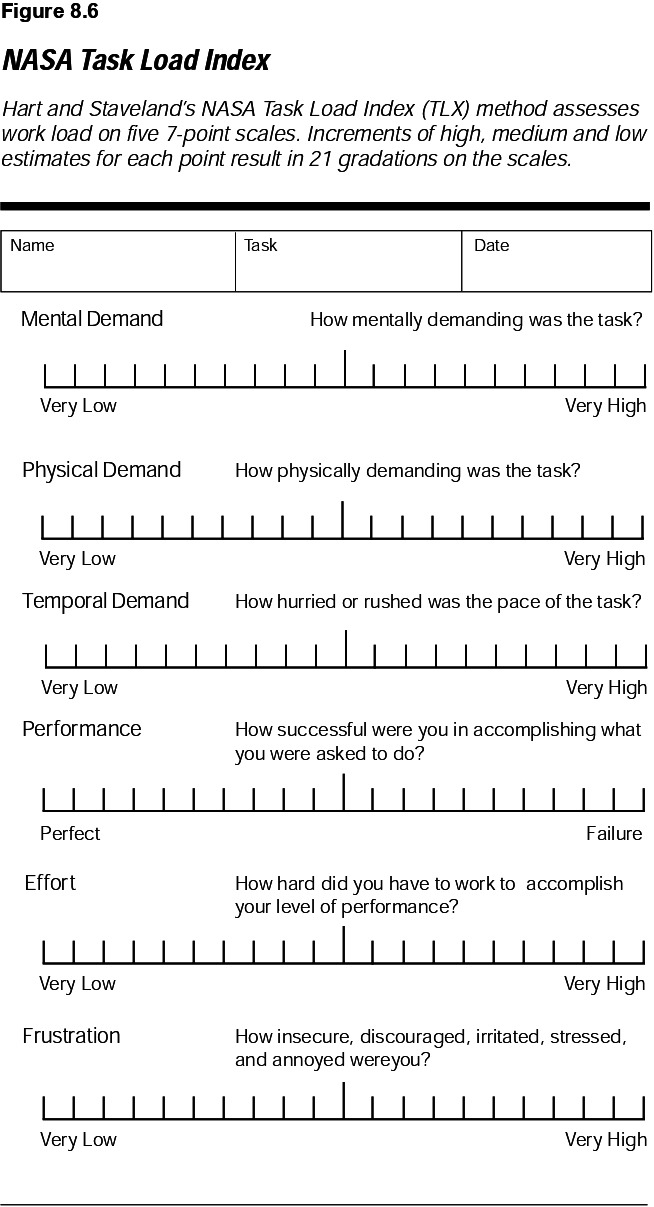
NASA Task Load Index Scale.

Overall workload will be assessed using the NASA Task Load Index (NASA-TLX). This quantitative scale assesses total workload over six domains (figure 3).^12^ The operator, assistant and scrub nurse will be asked to rate their mental, physical and temporal demand, as well as their performance, effort and frustration levels, on a 21-point scale. R-PCI and M-PCI survey scores will be compared overall, and within individual subdomains to identify any perceived differences in workload between the two treatment groups.

### Robotic/manual lesion and stent length comparison

We will be comparing robotic lesion and stent length estimates as well as visual lesion and stent length estimates performed by the operator. The operator will be asked to provide a visual estimation of expected lesion and stent length, this will be compared with the gold standard measurement determined by the robot. The robotic measurement will be performed as follows: the operator will advance a balloon past the lesion and position its proximal or distal marker at the distal end of the lesion. A digital counter located on the touchscreen (figure 1B) will then be zeroed. The operator will retract the balloon until the proximal end of the lesion is reached. The robotic lesion length measurement will be displayed by the digital counter.

### Follow-up

Patients will be followed up at day 3 or hospital discharge (whichever occurs first). Repeat pathology, including full blood count, renal function, troponin and creatine kinase myocardial band, will be collected and any clinical endpoints (as defined earlier) recorded. Further clinical follow-up will occur at day 30 and day 365, via telephone consultation. Any interim clinical endpoints will be recorded. These follow-ups will be conducted by a clinical trials coordinator specifically employed for the trial. Table 1 summarises the schedule for data collection and follow-ups.

**Table 1:** Schedule of data collection and clinical follow-up

	Screening	Index procedure	Day 3/discharge	Day 30	Day 365
Informed consent	✓				
Medical history, examination and vital signs	✓	✓			
Pathology and ECG	✓	✓	✓		
Randomisation and percutaneous coronary intervention procedure		✓			
Record adverse events		✓	✓	✓	✓
Complete case report form	✓	✓	✓	✓	✓

### PCI screening log

A deidentified screening log of all patients undergoing PCI within the cardiac catheterisation laboratories over the trial period will be maintained. Reasons as to why patients did or did not proceed to R-PCI will be recorded. General patient demographics and cardiovascular risk factors will be collected. This data will be used to determine patient, clinical or procedural predictors for patients not to undergo R-PCI.

### Radiation monitor comparison

Radiation exposure to the patient and the proceduralists will be recorded via two separate personal dosimeters systems, the Polimaster PM1610 X-ray and Gamma Radiation Personal Dosimeter and the RaySafe i3 Real-time Radiation Dosimeter. As mentioned in the Study endpoints section, the dosimeters will be positioned in standardised positions for the medical team and patient. For the proceduralists and assistants, the dosimeters will be worn outside the lead protection gowns, side by side, in a standardised position in the top pocket on the left side of the chest, over the heart. The dosimeter for the patient will be positioned at (or near) the left shoulder. The readings for the Polimaster and RaySafe dosimeters will be compared with each other (to assess agreement) as well as to the air kerma and dose area product readings recorded by the X-ray system.

### Statistical analysis

The sample size calculations are based on the primary endpoint of patient radiation exposure. A total sample of 300 patients (150 in each group) is sufficient to detect a 20% reduction in radiation exposure to the patient during PCI, assuming a mean radiation exposure of 1284 mGy with SD 180 mGy in the control group,^8^ and a SD of 142.83 mGy in the intervention group with >99% statistical power and two-sided statistical significance of 5%. A total sample size of 200 patients (100 in each group) will still provide>99% power to detect only a 10% reduction in radiation exposure to the patient during PCI. Study data will be collected and managed using Research Electronic Data Capture (REDCap) electronic data capture tool hosted and managed by Research Technology Services (University of New South Wales, Sydney, Australia).^13 14^ REDCap is a secure, password protected, web-based software platform designed to support data capture for research studies, providing (1) an intuitive interface for validated data capture; (2) audit trails for tracking data manipulation and export procedures; (3) automated export procedures for seamless data downloads to common statistical packages; and (4) procedures for data integration and interoperability with external sources. We have used a statistician to assist in the development of a randomisation schedule, which has been uploaded into REDCap. The clinical trials team will perform patient randomisation using its inbuilt programming.

Continuous variables will be summarised using the number of observations, mean, SD, median, minimum and maximum values. Categorical variables will be summarised with the number and percent in each category out of the number observed. Two-sided 95% CIs will be calculated and two-sided p values<0.05 will be considered statistically significant.

The outcome of patient radiation exposure will be analysed using analysis of variance. As each participant may have multiple lesions, the outcome of clinical success at day 3 will be analysed using a two level multilevel model. Logit link will be used with a binomial distribution, and restricted maximum likelihood will be used for estimation. Predictor variables in the models will include treatment group. Technical success will be analysed with Fisher’s exact test, comparing the proportion of participants with technical success between the two groups. Logistic regression will be used to compare technical success accounting for lesion complexity. For all other secondary outcomes, continuous outcomes will be measured using independent t-test (or non-parametric alternative depending on distributional assumptions), and categorical outcomes analysed by Fisher’s exact test. For outcomes measured repeatedly over time, multilevel models with appropriate link functions will be used for analysis. A Bland-Altman analysis will be performed to assess agreement between the two radiation dosimeter systems. All statistical analyses will be performed using SAS or other widely accepted statistical or graphical software. Trial data will be analysed on an intention to treat as well as a per-protocol basis.

### Safety monitoring

An independent data safety and monitoring adjudication board of interventional and non-interventional cardiologists has been established prior to commencement of the trial. They will review all clinical events in a blinded fashion. Adjudication will be based on narratives and source data. Source data that may be collected for review include, but are not limited to, deidentified angiograms, procedure reports, discharge summaries, laboratory results, follow-up consultations and preprocedure and postprocedure ECGs. The operating cardiologists may be contacted for queries and additional support documentation.

### Data management

All trial data will be collected by an authorised member of the research team and recorded onto standardised paper case report forms. This data will be transcribed and kept securely on REDCap. All participants’ medical and procedural information will be kept on a password-protected computer on a password accessed network drive within a locked office. Data from the trial will not be made available for review or sharing until after the completion of the trial and publications of results. A clinical audit of the trial and its data will be performed independently by an authorised research team member as well as the clinical trials coordinator at the end of the study period. All study documents will be retained for a minimum of 15 years after the completion of the study before being confidentially destroyed.

### Trial status and dissemination

The trial commenced enrolment in June 2023 and as of August 2024, a total of 114 patients have been recruited. Eight interventionalists have agreed to participate in the clinical trial and have completed the required clinical training. This consisted of a didactic lecture, simulator-based cases and then five proctored cases. This article presents the most up-to-date protocol (version 3), which was last updated in September 2023. Recruitment will conclude in December 2024 with follow-up to be completed by December 2025. Data analysis is expected to be completed by early 2026 with primary results published in mid 2026. The results of the trial will be published in peer-reviewed journals and presented at national and international conferences.

### Discussion

The hypotheses of the PARTY trial are that R-PCI, compared with M-PCI, will lead to less radiation exposure to the patient and operator with comparable safety and efficacy profiles. The initial results of studies investigating R-PCI have shown significant radiation exposure reduction to the operator, with high rates of efficacy and safety.^7 9^ There is also evidence of reduced radiation exposure to the patient.^8^ However, these studies have been either case series, observational or registry data. We hope the PARTY trial, which is to our knowledge the world’s first randomised controlled trial investigating R-PCI, will be able to provide robust evidence into the potential benefits of R-PCI.

We hypothesise that R-PCI will have additional advantages over M-PCI on top of the radiation reductions benefits. One potential benefit is reduced physical strain and fatigue to the operator due to the shortened duration that heavy lead protection equipment needs to be worn. The operator can perform the R-PCI case while seated within the shielded cockpit rather than standing at the patient’s bedside, improving ergonomics. The potential benefits of robotic assistance in reducing operator musculoskeletal strain and improving procedural precision have already been identified in surgery. A meta-analysis by Wee *et al* showed that among 29 articles and 3074 participants, robotic surgery, when compared with laparoscopic surgery, allowed for better ergonomics and reduced workload among operators.^15^ Furthermore, the authors identified lower self-reported physical discomfort among operators during robotic surgery. Shugaba *et al* combined the results of 10 studies and demonstrated that robotic surgery was associated with reduced electromyographic activation of the biceps, triceps, deltoid, trapezius and erector spinae muscles leading to reduced muscle fatigue.^16^ Moreover, the authors demonstrated improved cognitive load with robotic compared with laparoscopic surgery.^16^

We hypothesise that with the improved ergonomics of R-PCI discussed earlier, similar benefits to operator musculoskeletal strain and procedure precision can be identified. To investigate this, we will be assessing physical strain and overload workload via a workload assessment survey, which will be performed at the end of each case to determine the caseload demands on the operator, assistant and scrub nurse. The NASA-TLX has been tested in many industries including healthcare and is one of the most widely used (and independently validated) survey based workload measures.^17^

Another possible benefit of R-PCI over M-PCI is the precise movements and equipment stability provided by the machine. This may exceed the accuracy that could be achieved by the human hand (as the robot can accurately and reproducibly move the guidewires and intracoronary devices one millimetre if needed, via the touchscreen illustrated in figure 1B). This could potentially improve the overall accuracy and precision of lesion assessment and stent deployment, avoiding longitudinal geographical miss (LGM). This occurs when the deployed stent fails to completely cover the stenotic segment and may be associated with longer term morbidity and mortality consequences. These include increased risk of target lesion revascularisation and myocardial infarction,^18^ as well as the need for further stents to properly cover the lesion, increasing procedural costs, radiation and contrast usage as well as prolonging the PCI procedure. R-PCI has been shown to result in statistically significant lower rates of LGM compared with M-PCI.^18^ Using R-PCI is hypothesised to reduce the rates of LGM through several mechanisms, including increased accuracy of lesion measurements, improved intracoronary equipment delivery by the robot, better operator positioning in front of the X-ray screens for improved visualisation, and reduced operator fatigue due to being seated in the cockpit rather than prolonged standing at the bedside while wearing heavy lead gowns.^18^ In an analysis by Campbell *et al*, a cardiologist’s visual estimation of a lesion (and hence appropriate stent length to cover the lesion) was found to be too short in 32% of cases, too long in 33% of cases and accurate in only 35% of cases,^19^ when compared with the ‘gold standard’ robotic measurement.

### Conclusions

The randomised controlled PARTY trial will evaluate the safety, effectiveness including the clinical and technical performance and cost analysis of R-PCI versus M-PCI. This will be the first randomised controlled trial performed worldwide to investigate R-PCI with clinical follow-up of up to 1 year. The results of this trial will hopefully encourage the increased uptake of R-PCI in PCI.

## supplementary material

10.1136/openhrt-2024-002950 online supplemental file 1

## Data Availability

No data are available.
