# Supplementary material for: Rationale and design of the randomised, controlled Percutaneous coronary intervention using Assisted Robotic TechnologY (PARTY) trial
Source: Open Heart. 2024 Nov 21;11(2):e002950. doi: 10.1136/openhrt-2024-002950 (PMC11603688; doi:10.1136/openhrt-2024-002950)
Supplement: online supplemental file 1 [file openhrt-11-2-s001.docx]

**Percutaneous Coronary Intervention using assisted robotic technology:**

**a randomised controlled trial**

**Short title: PCI using Assisted Robotic TechnologY trial: the PARTY trial**

**Protocol Number: ING003**

**Coordinating Investigator:** **Craig Juergens**

**Sponsor: South Western Sydney Local Health District**

**Version Number: 3**

**Version Date: 19^th^ September 2023**

**Confidential Material:**

This document is the property of South Western Sydney Local Health District (SWSLHD) and is confidential and proprietary. The information contained herein is believed to be accurate and complete as of the date of preparation. The contents of this document may not be reproduced without prior expressed consent by SWSLHD.

**Table of Contents (need to edit this)**

[Corindus Randomised Controlled Trial Summary 6](#_Toc135811117)

[1.0 Introduction 9](#_Toc135811118)

[**1.1** **Device Name** 9](#_Toc135811119)

[**1.2** **Background** 9](#_Toc135811120)

[**1.3** **Risks & Benefits** 11](#_Toc135811121)

[1.3.1 Potential Risks 11](#_Toc135811122)

[1.3.2 Potential Benefits 11](#_Toc135811123)

[1.3.3 Assessment of Potential Risk & Benefit 12](#_Toc135811124)

[2.0 Objectives & Endpoints 12](#_Toc135811125)

[2.1 Primary Objective 12](#_Toc135811126)

[2.2 Effectiveness Performance Measures 12](#_Toc135811127)

[2.2.1 Clinical Success 12](#_Toc135811128)

[**2.3** **Technical Success** 13](#_Toc135811130)

[**2.4** **Safety Measures** 14](#_Toc135811131)

[2.4.1 Major Adverse Cardiac Events (MACE) 14](#_Toc135811132)

[2.4.2 Serious Adverse Events 14](#_Toc135811133)

[**2.5** **Cost Measures** 14](#_Toc135811134)

[**2.6** **Other Measures/ Procedural Characteristics** 14](#_Toc135811135)

[2.6.1 Overall Procedure Time Measure 14](#_Toc135811136)

[2.6.2 PCI Procedure Time 14](#_Toc135811137)

[2.6.3 Wiring Time 15](#_Toc135811138)

[2.6.4 Fluoroscopy Time 15](#_Toc135811139)

[2.6.5 Participant and Staff Radiation Exposure 15](#_Toc135811140)

[2.6.6 Contrast Fluid Volume 15](#_Toc135811141)

[2.6.7 Equipment utilization (stents, wires, balloons, catheters) 15](#_Toc135811142)

[All equipment used in the PCI will be recorded to perform a cost analysis of technology-assisted PCI versus standard PCI performed by a cardiologist. 15](#_Toc135811143)

[**2.7** **Lesion Assessment** 15](#_Toc135811144)

[**2.8** **Workload assessment survey** 15](#_Toc135811145)

[3.0 Study Design 16](#_Toc135811146)

[**3.1** **Overview** 16](#_Toc135811147)

[**3.2** **Schema** 17](#_Toc135811148)

[**3.3** **Schedule of Events** 18](#_Toc135811149)

[**3.4** **End of Study Definition** 18](#_Toc135811150)

[4.0 Study Population 19](#_Toc135811151)

[**4.1** **Inclusion Criteria** 19](#_Toc135811152)

[**4.2** **Exclusion Criteria** 19](#_Toc135811153)

[**4.3** **Screen Failure** 19](#_Toc135811154)

[**4.4** **Registry of PCIs** 19](#_Toc135811155)

[**4.5** **Strategy for Recruitment and Retention** 19](#_Toc135811156)

[5.0 Study Intervention 20](#_Toc135811157)

[**5.1** **CorPath GRX** 20](#_Toc135811158)

[5.1.1 Intended Use & Device Description 20](#_Toc135811159)

[5.1.2 CorPath GRX Remote Workspace 20](#_Toc135811160)

[5.1.3 CorPath Bedside Unit 21](#_Toc135811161)

[**5.2** **Storage and Accountability** 21](#_Toc135811162)

[**5.3** **Randomisation** 22](#_Toc135811163)

[**5.4** **Concomitant Therapy** 22](#_Toc135811164)

[6.0 Study Intervention Discontinuation and Participant Discontinuation/ Withdrawal 22](#_Toc135811165)

[**6.1** **Participant Withdrawal** 22](#_Toc135811166)

[**6.2** **Lost to follow up** 23](#_Toc135811167)

[7.0 Study Assessment and Procedures 23](#_Toc135811168)

[**7.1** **Screening Assessment** 24](#_Toc135811169)

[**7.2** **Index Procedure (PCI)** 24](#_Toc135811170)

[7.2.1 Peri-Procedure Study Medication Regimen 24](#_Toc135811171)

[7.2.2 Data to be collected prior to initiating PCI 24](#_Toc135811172)

[7.2.3 Data to be collected during the procedure 24](#_Toc135811173)

[7.2.4 Standard PCI Technique 25](#_Toc135811174)

[7.2.5 CorPath Use Technique 25](#_Toc135811175)

[7.2.6 Conversion to Manual Procedure Data Collection 26](#_Toc135811176)

[7.2.7 Partial Robotic Assistance Data Collection 27](#_Toc135811177)

[7.2.8 Post-Procedure Data Collection 27](#_Toc135811178)

[**7.3** **Day 3 or Discharge** 27](#_Toc135811179)

[**7.4** **Day only PCI patients** 28](#_Toc135811180)

[**7.5** **Day 30** 28](#_Toc135811181)

[**7.6** **Day 365 or End of Study (EOS)** 28](#_Toc135811182)

[**7.7** **Adverse Events, Event Assessment and Serious Adverse Device Events** 28](#_Toc135811183)

[7.7.1 Adverse Events (AEs) 28](#_Toc135811184)

[7.7.2 Event Assessments 29](#_Toc135811185)

[7.7.3 Serious Adverse Device Effect (SADE) 30](#_Toc135811186)

[7.7.4 Event Relationships 31](#_Toc135811187)

[7.7.5 Event Expectedness 31](#_Toc135811188)

[7.7.6 Event Severity 31](#_Toc135811189)

[7.7.7 Device Deficiency 32](#_Toc135811190)

[**7.8** **Reporting Procedures** 32](#_Toc135811191)

[7.8.1 General Reporting Requirements (AEs and Device Deficiencies) 32](#_Toc135811192)

[7.8.2 Reporting Requirements of SADEs 32](#_Toc135811193)

[8.0 Data Management, Statistical Analysis and Record Keeping 32](#_Toc135811194)

[**8.1** **Statistical Methods** 33](#_Toc135811195)

[**8.2** **Data Management** 34](#_Toc135811196)

[**8.3** **Study Data Requirements** 34](#_Toc135811197)

[**8.4** **Data Entry** 34](#_Toc135811198)

[**8.5** **Final Data Analyses** 34](#_Toc135811199)

[**8.6** **Data Retention** 34](#_Toc135811200)

[9.0 Monitoring 35](#_Toc135811201)

[**9.1** **Protocol Training** 35](#_Toc135811202)

[**9.2** **Device Training** 35](#_Toc135811203)

[**9.3** **Confidentiality and Protection of Study Files** 35](#_Toc135811204)

[10.0 Data Quality Assurance 35](#_Toc135811205)

[**10.1** **Clinical Event Handling** 36](#_Toc135811206)

[**10.2** **Study Endpoint Event Adjudication** 36](#_Toc135811207)

[11.0 Administrative Responsibilities 36](#_Toc135811208)

[**11.1** **Human Research Ethics Committee (HREC)** 36](#_Toc135811209)

[**11.2** **Informed Consent Procedures** 37](#_Toc135811210)

[**11.3** **Confidentiality** 37](#_Toc135811211)

[**11.4** **Study Registration and Publication Policy** 38](#_Toc135811212)

[12.0 References 38](#_Toc135811213)

[APPENDIX A. DEFINITIONS 41](#_Toc135811214)

**Protocol Signature Page**

I have read and understand the contents of this protocol. I agree to follow and abide by the guidelines set forth in this document.

Investigator Name (print)

Investigator Signature Date

# Corindus Randomised Controlled Trial Summary

| **Title:** | Percutaneous Coronary Intervention using assisted robotic technology: a randomised controlled trial |
| --- | --- |
|  |  |
| **Short Title:** | **P**CI using **A**ssisted **R**obotic **T**echnolog**Y** trial: the **PARTY** trial |
|  |  |
| **Study Description:** | Coronary artery disease is a leading cause of death globally, nationally and locally. Currently in South Western Sydney Local Health District Interventional Cardiologists perform Percutaneous Coronary intervention (PCI) procedure which opens coronary arteries that are narrowed or blocked by the build-up of atherosclerotic plaque. PCI may also be used to relieve symptoms of coronary heart disease or to reduce heart damage during or after a heart attack.  With new technology, engineering and training development, a technology-assisted percutaneous coronary intervention (PCI) device has the potential to increase procedural accuracy, reduce radiation exposure to participants and staff, improved ergonomics, and reduce major adverse cardiac events. To date we are not aware of any randomised controlled trial data supporting this technology |
| **Study Design:** | This is a randomized controlled trial of the CorPath GRX System versus standard PCI to compare participant outcomes at 72 hours post-procedure or hospital discharge, day 30 and 1 year. |
|  |  |
| **Study Objective:** | This study will assess the safety, effectiveness including the clinical and technical performance and cost analysis of technology-assisted PCI versus standard PCI performed by a cardiologist in a randomised controlled trial. |
| **Effectiveness Performance Measures:** | **Clinical Success:**  Less than 30% residual stenosis (visual estimate) post PCI, without in-hospital major adverse coronary events (MACE).  **Technical Success:**  Defined as successful completion of the PCI absent ***unplanned*** conversion to manual operation of guidewires or balloon/stent catheter or inability to navigate vessel anatomy or poor guide catheter support. |
|  |  |
| **Safety Measures:** | **Major Adverse Cardiac Event (MACE):**  MACE that occurs within 72 hours of the procedure or prior to hospital discharge, whichever occurs first, day 30 and 1 year  **Serious Adverse Events:**  All Serious Adverse Events (SAEs) from the start of the PCI procedure until the end of the study will be summarized. |
| **Cost Measures:** | Total procedural cost of PCI at day 30 and 1 year |
| **Other Measures/Procedural Characteristics:** | **Overall Procedure Time**  Defined as the time measured from the insertion of the hemostasis sheath until the removal of the guide catheter.  **PCI Procedure Time -** Defined as the time measured from the insertion of the guide catheter until the removal of the guide catheter.  **Fluoroscopy Time –** total fluoroscopy utilized during the procedure as recorded by an Imaging System.  **Participant Radiation Exposure** – DAP (dose-area-product) and cumulative dose/air kerma as recorded during the procedure.  **Staff Radiation Exposure** – DAP (dose-area-product) and cumulative dose/air kerma as recorded during the procedure.  **Contrast Fluid Volume in mLs**  **Equipment utilization**  **Operator and staff survey of workload**  **Measurement of Lesion and Stented Segment Length**  Compare (1):   - Visual estimate of segment to be stented by clinical operator prior to delivery of PCI device(s). - CorPath GRX System measurement of the segment planned for treatment with stent. |
|  | Compare (2):   - CorPath GRX System measurement of stent length with known length of stented segment |
|  |  |
| **Participant Population:** | Participants aged 18-85 years with evidence of coronary artery disease and with a clinical indication for PCI will be asked to participate in this study. Planned recruitment of 150 participants per study arm over 3 years. |
|  |  |
| **Inclusion Criteria:** | In order to be eligible to participate in this study the individual must meet all of the following criteria:   1. Age ≥18 years- 85 years 2. Participants with coronary artery disease with clinical indication for PCI 3. Participant deemed appropriate for robotic-assisted PCI 4. Provided a signed and dated informed consent form. |
|  |  |
| **Exclusion Criteria:** | An individual who meets any of the following criteria will be excluded from participation in this study:   1. The investigator determines that the participant or the coronary anatomy is not suitable for robotic-assisted PCI. |
|  |  |
| **Investigational Sites:** | Participants will be enrolled from South Western Sydney Local health District and referred to Liverpool Hospital for the PCI procedure. |
|  |  |
|  |  |
| **Principal Investigator:** | Craig Juergens, MBBS, D Med Sc, FRACP FACC  Director of Medicine/Interventional Cardiologist  Liverpool Hospital, NSW Australia University of NSW, Sydney |
|  |  |
| **Device:** | **CorPath GRX System:**  Bedside Unit consists of the Extended Reach Arm, Robotic Drive and single-use Cassette.  Remote Workstation consists of the Interventional Cockpit and the Control Console. |
|  |  |
| **Indication for Use:** | The CorPath GRX is intended for use in the remote delivery and manipulation of guidewires and rapid exchange balloon/stent catheters, and remote manipulation of guide catheters during percutaneous coronary intervention (PCI) procedures. |
|  |  |
| **Manufacturer:** | Corindus Vascular Robotics  309 Waverley Oaks Road, Suite 105  Waltham, MA 02452 |
|  |  |

# Introduction

Coronary artery disease is a leading cause of death globally, nationally and locally. Currently in South Western Sydney Local Health District, Interventional Cardiologists perform Percutaneous Coronary intervention (PCI) procedure which opens coronary arteries that are narrowed or blocked by the build-up of atherosclerotic plaque. PCI may also be used to relieve symptoms of coronary heart disease or to reduce heart damage during or after a heart attack.  With new technology, engineering and training development, a technology-assisted percutaneous coronary intervention (PCI) device has the potential to increased procedural accuracy, reduce radiation exposure to participants and staff, improved ergonomics and reduce major adverse cardiac events. To date we are not aware of any randomised controlled trial data supporting this technology.

## **Device Name**

CorPath^®^ GRX System

## **Background**

Until the introduction of the CorPath^®^ 200 System into the market, all coronary interventional procedures were performed under fluoroscopic guidance with the medical team standing near the participant while maneuvering the devices manually. The interventional cardiologist and the medical team are routinely exposed to ionizing radiation [1-3]. The consequences of long-term radiation exposure, even at low doses, are meaningful and may be associated with cancer [4-12], cataracts [[3](#_ENREF_5), 13-15] and other medical problems [16, 17]. To minimize the radiation exposure, the staff is protected with heavy lead aprons and other protection devices [18 - 22]. In spite of these protective measures, the team is still exposed to radiation and therefore undergoes careful follow-up and testing for prevention of overexposure [[23](#_ENREF_24)]. While the conventional protective methods are useful to minimize radiation exposure, they create an uncomfortable working environment for the operator, who must perform precise work under a physically strenuous environment for prolonged periods of time, leading to orthopedic hazards of the profession [[24](#_ENREF_25) ,25].

CorPath is the only robotic-assisted platform cleared for percutaneous coronary intervention (PCI). Since the FDA clearance of the CorPath 200 System in 2012, operators have performed over 2000 robotic PCI procedures. Feedback indicates the System is user-friendly, offers enhanced visualization for precise stent placement and allows for a safer work environment. However, with iterative changes in technology, the CorPath 200 has been enhanced by improvements in workflow and remote guide catheter control.

The CorPath GRX System is the second generation of the CorPath platform. With CorPath GRX, workflow is streamlined by the addition of an easily positioned extended reach arm. The new arm incorporates a "touchscreen". The benefits of the touchscreen allow the tableside user to visualize the instructions. Workflow enhancements also include a redesigned cassette to allow for active guide management. The clinical improvements consist of the addition of Active Guide Management, faster guidewire rotation and improved measurement accuracy. These improvements have the potential to allow for greater robotic procedural control for the primary operator and, perhaps a reduction in not only operator but also participant and Cath Lab team radiation exposure.

One of the key differentiators between the first and second generation of CorPath is the ability to robotically manipulate the guide catheter using Active Guide Management. Using the joystick or alternatively the touchscreen, you can advance and/or rotate the guide catheter back into position.

Since all CorPath cases start with manual insertion of a guiding catheter, it is critical to have the guiding catheter seated correctly in the coronary ostium. For optimal robotic guidewire/balloon/stent delivery, adequate guide catheter support (also known as *backup support*) is necessary. Adequate guide catheter support revolves around the ability of the guide catheter to remain in position and to provide appropriate stability for the advancement of interventional equipment [26]. This guide catheter support enables the robotic procedure to progress. Sometimes, as the physician attempts to advance the guidewire or balloon/stent robotically, resistance in movement occurs causing the guide catheter to disengage from the coronary ostium. The CorPath 200 does not allow for any guide control manipulation from the remote cockpit. The physician must either leave the cockpit and adjust the guide catheter manually, or passively reengage the catheter by retracting the guidewire, to allow for proper alignment and reengagement in the coronary ostium. Having to leave the cockpit for this reason increases radiation exposure to the physician. Only after manual adjustment of the guide catheter can the CorPath case continue.

This has been addressed in the design of CorPath GRX, the next generation CorPath platform. The CorPath GRX System offers Active Guide Management which is incorporated into the re-designed cassette and radiation-shielded cockpit.

## **Risks & Benefits**

This purpose of this study is to assess the safety, effectiveness including the clinical and technical performance and cost analysis of technology-assisted PCI versus standard PCI performed by a cardiologist in the context of a randomised controlled trial. The potential risk and benefits are listed below.

### 1.3.1 Potential Risks

Participants may be exposed to potential risks from participating in this study. Potential procedure and device related adverse events that may occur and/or require intervention are described below. The primary risks of the procedure are believed to be similar to the risks of participants undergoing procedures requiring PCI. Complications may occur at any time during the procedure. Potential risks of the PCI are:

- Injury to the hearts arteries including tearing and rupture
- Infection, bleeding and bruising at the catheter site
- Allergic reaction or kidney damage due to the dye or contrast used
- Blood clots that can lead to stroke or heart attack
- Bleeding into the abdomen

This study involves exposure to a small amount of radiation during the coronary angiogram which the participant would be receiving as standard care. Radiation may slightly elevate the risk for cancer. There is no increase in risk of exposure as a result of participating in this study.

Complications may occur at any time during the procedure. The study may involve unknown or unforeseen side effects or complications other than those mentioned above.

### 1.3.2 Potential Benefits

The CorPath System has shown clinical success in approximately 10,000 cases worldwide. The Precise Study demonstrated a 97.6% clinical success of all treated lesion (27) . A recent study showed that there is a reduction of unnecessary additional stents by 8.3% (28).

Radiation safety in the Interventional suite is also seen to be decreased with the use of the CorPath System by 20% for participants (29) and 95% to the primary operator (27).

There is limited real world evidence of the cost effectiveness of standard PCI versus technology assisted PCI. This study will help us to address this.

There may be other benefits that are unforeseen at this time.

### 1.3.3 Assessment of Potential Risk & Benefit

PCI is a standard care for Coronary Artery Disease (CAD). The complications of the procedure are explained to the participants prior to PCI and are similar in both arms of the study. The CorPath System is registered on the Australian Registry Therapeutic Goods (ARTG) for use in the remote delivery and manipulation of devices during percutaneous coronary and vascular procedures, including Guidewires, Rapid Exchange Catheters, Guide Catheters, Microcatheters, Neurovascular, Stent Retrievers, Embolization Coils and Coil Assist Stents. This study will use the device within indication.

The risk of collecting the data to compare two approved procedures has therefore been assessed as being low given that strict data protection is in place.

# Objectives & Endpoints

# Primary Objective

The primary objective of this study is assess the safety, effectiveness including the clinical and technical performance and cost analysis of technology-assisted PCI versus standard PCI performed by a cardiologist in a randomized clinical trial.

# Effectiveness Performance Measures

The following study measures will be evaluated in participants enrolled in this clinical trial. Parameters are participant-based or lesion-based.

### 2.2.1 Clinical Success

Defined as less than 30% residual stenosis (visual estimate) post PCI in the lesion(s) treated, without in-hospital MACE.

MACE is defined as cardiac death, clinically relevant MI after coronary revascularization (Q-wave or non-Q-wave myocardial infarction), or clinically driven target vessel revascularization (TVR) by PCI or CABG. Clinically relevant MI after coronary revascularization will be defined by the 4^th^ Universal Definition of PCI related MI. Events as classified by the Society for Cardiovascular Angiography and Interventions definition for periprocedural MI will also be recorded for additional analysis.

The 4th Universal Definition of PCI related MI defines:

- Participants with normal baseline cardiac biomarkers
  - Cardiac troponin (cTN) >5x the 99^th^ percentile of upper reference limit (URL)
- Participants with elevated baseline cardiac biomarkers
  - Rise in post procedure cTN by >20% and be at least >5x 99^th^ percentile of URL AND
  - At least one of the following
    - New ischaemic ECG changes
    - Development of new pathological Q waves
    - Imaging evidence of new loss of viable myocardium or new regional wall motion abnormality in a pattern consistent with an ischaemic aetiology
    - Angiographic findings consistent with a procedural flow limiting complication such as coronary dissection, occlusion of a major epicardial artery or a side branch occlusion/thrombus, disruption or collateral flow or distal embolisation

The Society for Cardiovascular Angiography and Interventions definition for periprocedural MI defines:

- Participants with normal baseline cardiac biomarkers
  - CK-MB >10x upper limit normal (ULN) or cTn (I or T) >70x ULN, or
  - CK-MB >5x ULN or cTn >35x ULN plus new Q-waves in >2 contiguous leads or LBBB
- Participants with elevated baseline cardiac biomarkers
  - Baseline biomarkers are stable or falling:
    - CK-MB >10x ULN or cTn (I or T) >70x ULN of **most recent pre-procedure level**, or
    - CK-MB >5x ULN or cTn >35x ULN of **most recent pre-procedure level** plus new Q-waves in >2 contiguous leads or LBBB
  - Baseline biomarkers have not been shown to be stable or falling: CK-MB (or cTn) rises by an absolute increment equal to those recommended above plus new ST-segment elevation or depression plus signs consistent with a clinically relevant MI, such as new onset or worsening heart failure or sustained hypotension.

## **Technical Success**

Defined as successful completion of the PCI absent *unplanned* conversion to manual operation of guidewire or balloon/stent catheter or inability to navigate vessel anatomy or poor guide catheter support.

## **Safety Measures**

Several safety-based measures will also be utilized to confirm the overall safety of procedures involving the CorPath GRX System and standard of care. These measures include:

### Major Adverse Cardiac Events (MACE)

MACE will be measured at each visit of the study.

### Serious Adverse Events

All Serious Adverse Events (SAEs) from the start of procedure until the end of the study will be summarized.

## **Cost Measures**

The cost of standard PCI versus technology-assisted PCI will be measured using equipment utilisation and post procedural length of stay until discharge. The costs of these two measurements will be compared and summarized.

## **Other Measures/ Procedural Characteristics**

The objective of this study is to compare standard PCI with technology-assisted PCI to collect real-world data. When the PCI is being performed in conjunction with a diagnostic procedure, all efforts should be made to isolate the PCI procedural characteristics from the diagnostic procedure. At the end of the diagnostic procedure or alternatively to coincide with the PCI procedure time record the contrast, fluoroscopy time, DAP & AK at the end of the diagnostic procedure. When the PCI procedure is finished, record the values for these characteristics accumulated during the PCI procedure and not the diagnostic procedure. Procedures will be identified as staged (PCI only) or ad-hoc (LHC possible PCI). Any requirement to move from technology-assisted PCI to standard care will also be captured.

### Overall Procedure Time Measure

Defined as the time measured from the insertion of the hemostasis sheath until the removal of the guide catheter

### PCI Procedure Time

Defined as the time measured from the insertion of the guide catheter until removal of the guide catheter.

### Wiring Time

Defined as time measured from advancement of coronary wire out of guide catheter until it crosses the lesion

### Fluoroscopy Time

Total fluoroscopy utilized during the PCI procedure, as recorded by an imaging system.

### Participant and Staff Radiation Exposure

DAP (dose-area-product) and cumulative dose/air kerma, as recorded during the PCI procedure. Radiation exposure to the patient and staff will be measured by personal dosimeters (Polimaster PM1610) placed at the left shoulder.

### Contrast Fluid Volume

Total volume used during the PCI procedure in mLs.

### Equipment utilization (stents, wires, balloons, catheters)

### All equipment used in the PCI will be recorded to perform a cost analysis of technology-assisted PCI versus standard PCI performed by a cardiologist.

## **Lesion Assessment**

The lesion will be measured and recorded by the Cardiologist during the PCI. During the standard care PCI the Cardiologist will measure the lesion as per the institutional procedures. For the technology-assisted PCI the lesion length will be measured with the CorPath GRX System as per the Operations Manual.

The physician will attempt to use the same measuring technique for both pre and post lesion measurements (treatment length and stent length).

A comparison on lesion and PCI device length will be performed:

- 1. Length of segment needing intervention: visual estimate vs. CorPath GRX measurement.
  2. Length of stent(s): manufacturer’s labeled stent length(s) vs. CorPath GRX measurement of implanted stent length(s)

## **Workload assessment survey**

The operator, assistant and nurse involved with the PCI procedure will be asked to complete a survey at the end of the procedure to record an assessment of their workload. This will be via the NASA Task Load Index, which is a survey which assesses workload over 6 domains (mental, physical and temporal demand) [30]. Initially designed for the aerospace industry and NASA, it has been widely utilised and validated across multiple industries [31], including the medical field. It is the most widely cited survey based workload measure [32]

# Study Design

## **Overview**

This is a randomized controlled trial of the CorPath GRX System versus standard PCI to compare participant outcomes at 72 hours post-procedure or hospital discharge, day 30 and 1 year.

The study population will consist of all consecutive participants undergoing PCI at Liverpool Hospital being asked to provide written informed consent prior to PCI and meet the eligibility criteria.

The study will randomize 300 participants equally into the 2 arms:

- Arm 1: PCI using the CorPath GRX System
- Arm 2: PCI using standard care

All enrolled participants will be followed post procedure through hospital discharge or 72 hours, whichever occurs first. The follow up will occur at day 30 and 1 year post-procedure

## **Schema**

Visit 1

Total 300: Perform screening assessment: Obtain written informed consent. Screen potential participants by inclusion and exclusion criteria; obtain history, protocol requirements and document.

Screening

Randomize

Confirm consent

Refer to **Section 3.3 Schedule of Activities**

Perform PCI

Visit 2

Index

Procedure

Visit 3

Refer to **Section 3.3 Schedule of Activities**

Participant discharged

Day 3/Discharge

Visit 4

Follow-up assessments of study endpoints

Refer to **Section 3.3 Schedule of Activities**

Day 30

**Final Assessments**

Refer to **Section 3.3 Schedule of Activities**

Visit 5

End Of Study

## **Schedule of Events**

| **Exam/ Test/ Data Collection** | **Screening** | **Index**  **Procedure** | **Day 3/ discharge** | **Day 30**  **(±5 days)** | **EOS / Day 365**  **(±14 days)** |
| --- | --- | --- | --- | --- | --- |
| Informed consent | X | X |  |  |  |
| Medical history | X |  |  |  |  |
| Demographics | X |  |  |  |  |
| Vital signs including height, weight^1^ | X | X^6^ | X^6^ |  |  |
| Physical examination | X^1^ |  | X |  |  |
| 12 lead ECG | X^4^ |  | X |  |  |
| non-cardiac laboratory assessments | X |  | X |  |  |
| Cardiac enzymes | X^2^ |  | X^3^ |  |  |
| Inclusion/ exclusion criteria | X |  |  |  |  |
| Randomisation |  | X |  |  |  |
| PCI |  | X |  |  |  |
| Dosimeter reading |  | X |  |  |  |
| Fluoroscopy reading |  | X |  |  |  |
| Lesion assessment |  | x |  |  |  |
| Time of procedure |  | X |  |  |  |
| Contrast usage |  | X |  |  |  |
| Equipment usage |  | X |  |  |  |
| Concomitant medications | X | X | X | X | X |
| Adverse Events^5^ |  | X |  |  |  |
| Health care resource usage |  | X | X | X | X |
| Event Assessment |  | X | X^5^ | X^5^ | X^5^ |
| Complete Case Report Form | X | X | X | X | X |

^1^ Within 30 days of index procedure

^2^ Pre-procedure cardiac enzymes within 7 days of index procedure, if collected.

^3^ Post-procedure cardiac enzyme collection is left to the discretion of the investigator and shall be dictated by the participant’s clinical symptoms.

^4^ Post-procedure 12-lead ECG: it is recommended that an ECG be performed at the time of discharge but is not required for the protocol.

^5^ Related to procedure, intervention or Adverse Event of Special Interest (MACE)

^6^ height and weight only required at screening

## **End of Study Definition**

A participant is considered to have completed the study if he or she has completed all phases of the study including the last visit or the last scheduled procedure shown in the Schedule of Activities.

The end of the study is defined as completion of the last visit or procedure shown in the Schedule of Activities in the trial.

# Study Population

Participants with coronary artery disease with a clinical indication for PCI and meet the below eligibility criteria will be enrolled into the study.

## **Inclusion Criteria**

- Age 18 to 85 years
- Participants with coronary artery disease with clinical indication for PCI
- Participant deemed appropriate for robotic-assisted PCI
- Able to provide a signed and dated informed consent form

## **Exclusion Criteria**

- The investigator determines that the participant or the coronary anatomy is not suitable for robotic-assisted PCI.

## **Screen Failure**

Screen failures are defined as participants who consent to participate in the clinical trial but are not subsequently randomly assigned to the PCI or entered in the study. All data collected at the screening visit or prior to the time of consent withdrawal will be included in the analysis.

## **Registry of PCIs**

A deidentified registry of all patients undergoing PCI within the Liverpool Hospital cardiac catheterisation lab over the trial period will be kept. Reasons as to why patients did or did not proceed to R-PCI will be recorded.

## **CT and Coronary Angiography Correlation**

A subset of 20 patients undergoing coronary angiogram ± PCI, who have had CT coronary angiograms as part of their workup, will have their anonymised angiogram and CT images compared and correlated by Siemens to improve image quality. The anonymised data will be stored on an encrypted hard drive and then transferred to Siemen’s IT department in Germany using a password protected secure file transfer platform. The data will be stored securely within the Siemens IT server and will be deleted upon completion of the analysis.

## **Strategy for Recruitment and Retention**

All participants who are presenting to Liverpool Hospital for angiography and potential PCI will be asked to participate in this study. Participants will be consented according to Section 11.2 of the protocol.

After the participant has provided consent, the Investigator will confirm eligibility against the inclusion and exclusion criteria. If the participant meets the criteria they will be randomized according to Section 5.3 of the protocol. The participant and their significant others will be blinded to the method of PCI.

Participants will be asked to return for study visits as identified in Section 7. The participant will be contacted prior to the visit to confirm the appointment. If they are unable to attend all efforts will be made to collect the measurements as per the protocol.

If participants withdraws from the study please refer to Section 6.

# Study Intervention

Participants will be randomized to PCI using assisted robotic technology using the CorPath GRX or standard PCI.

## **CorPath GRX**

### Intended Use & Device Description

The CorPath GRX is intended for use in the remote delivery and manipulation of guidewires and rapid exchange balloon/stent catheters, and remote manipulation of guide catheters during percutaneous coronary intervention (PCI) procedures.

The CorPath GRX System is designed to allow the interventional cardiologist to remotely manipulate guidewires, rapid exchange balloon/stent catheters and guide catheters within a participant’s vasculature to perform PCI procedures in a precise and well-controlled fashion, without being exposed to radiation levels typically encountered during a conventional (i.e., manual) PCI. The CorPath GRX System is composed of a Remote Workspace and a Bedside Unit.

### CorPath GRX Remote Workspace

The Remote Workspace consists of a radiation-shielded Interventional Cockpit that houses the Control Console. The Remote Workspace is also designed to integrate a power vision monitor as well as other manufacturers’ angiographic and hemodynamic video monitors, to afford the operator improved visualization of the PCI procedure.

The Control Console component has a touch-screen and three joysticks (one joystick for balloon/stent manipulation, one joystick for guidewire manipulation and one joystick for guide catheter manipulation). The balloon/stent joystick allows for precise control of linear motion (advancement and retrieval) of its respective devices. The guidewire joystick allows for both linear and rotational movement (clockwise and counterclockwise) of the guidewire. The guide catheter joystick allows for precise control of linear motion (advancement and retrieval) and for rotational movement (clockwise and counterclockwise) of the guide catheter. The devices are controlled independently, which allows operations to be performed individually (by using one joystick at a time) or simultaneously (by activating multiple joysticks at once). For precise, discrete manipulation, the balloon/stent, guidewire and guide catheter can also be manipulated in discreet 1-mm increments via the touch-screen buttons on the Control Console.

### CorPath Bedside Unit

The Bedside Unit consists of three components: (1) Extended Reach Arm, (2) Robotic Drive and (3) single-use Cassette (“Cassette”). The Extended Reach Arm supports the Robotic Drive, which houses the Cassette. The joysticks on the Control Console remotely deliver signals through a communication cable to the Robotic Drive that operates the Cassette. After the selected guidewire and catheter are loaded into the Cassette, the Cassette translates the signals from the joystick manipulations into the linear and rotational movements of the guidewire and guide catheter and the linear movements of the balloon/stent. The Cassette has a Y-connector holder which holds the guide catheter hub and is attached to the drive gear and CoPilot. The drive gear enables guide catheter rotation.

The CorPath GRX System sub-units function together via a Communication Cable. The CorPath GRX System utilizes proprietary software that is designed to operate within the parameters that have been proven to execute precise motion and movement of guidewires, balloon/stent systems and guide catheters within a blood vessel.

***Note: For a more detailed description of the CorPath GRX please refer to the CorPath GRX System Operator’s Manual.***

## **Storage and Accountability**

The investigator is responsible for device accountability at the study site. The investigator may delegate some of the investigator’s duties for device accountability at that site to an appropriate staff member. All devices provided by the Ingham Institute for Applied Medical Research will be stored in a secure and locked area at the site.

The investigator must maintain records that document device delivery to the study site, the inventory at the site, administration to each participant, and submit required forms to the appropriate authority if requested. These records include at a minimum date (received, used, returned), quantities, lot/serial numbers, and expiration dates assigned to the study device and the participant’s identification code, if used. Study devices must be recorded on the Device Accountability Log. The investigator must notify the appropriate authority of any damaged or unusable devices that were supplied to the site.

## **Randomisation**

Participants will be randomized 1:1 to the PCI performed using standard care or technology-assisted PCI in blocks to ensure balanced distribution of the treatment groups at all times. The study team will enter the participant screening information into the electronic data capture (EDC) system after informed consent has been obtained. Once the participant is determined to be eligible prior to the Index Procedure the study team will randomise the participant using the EDC. The Investigator will be advised of this and the allocated method of PCI will be performed. The participant will ideally remain blinded to the method of PCI performed.

## **Concomitant Therapy**

Medications to be reported in the Case Report Form (CRF) are concomitant prescription medications, over-the-counter medications and supplements.

# Study Intervention Discontinuation and Participant Discontinuation/ Withdrawal

In the event that the participant is randomized but does not go ahead to have PCI or there is a requirement to move from technology-assisted PCI to manual PCI they will not be discontinued from the study and the remaining study procedures should be completed as indicated by the study protocol. Any discontinuation of the participant after the PCI is described in Section 6.1.

## **Participant Withdrawal**

Once the participant has been enrolled in the study, s/he may withdraw consent to participate in the study at any time without prejudice. Participation in this clinical investigation is entirely voluntary. Likewise, there may be a reason identified by the Investigator that deems the participant no longer suitable for the study. If participant safety is not at risk, all reasonable efforts should be made to retain a participant prior to consideration for withdrawal as this can significantly impact data quality. In either case, the Investigator will contact the appropriate authority to discuss the circumstances for discontinuation/withdrawal. Discontinuation or withdrawal may occur for any of the reasons listed below (this list is not all inclusive).

- Participant is uncooperative with compliance of required study tests, medical management and/or procedures
- Investigator determines that participant has developed a condition in which continued participation in the study is considered potentially harmful to the participant
- Participant withdraws their consent
- Participant is lost to follow-up
- Participant has a significant protocol violation
- Participant incorrectly enrolled in the study
- the appropriate authority terminates the study

## **Lost to follow up**

A participant will be considered lost to follow-up if he or she fails to return for scheduled visits and is unable to be contacted by the study site staff.

The following actions must be taken if a participant fails to return to the clinic for a required study visit:

- The site will review the Electronic Medical Records (EMR) and/or contact the referring Cardiologist to determine if the participant is alive.
- The site will attempt to contact the participant and reschedule the missed visit ( within 1 month) and counsel the participant on the importance of maintaining the assigned visit schedule and ascertain if the participant wishes to and/or should continue in the study.
- Before a participant is deemed lost to follow-up, the investigator or designee will make every effort to regain contact with the participant (where possible, 3 telephone calls and, if necessary, a certified letter to the participant’s last known mailing address or local equivalent methods). These contact attempts should be documented in the participant’s medical record or study file.
- Should the participant continue to be unreachable, he or she will be considered to have withdrawn from the study with a primary reason of lost to follow-up.

# Study Assessment and Procedures

## **Screening Assessment**

The following evaluations are to be performed up to 30 days prior to the PCI

- Obtain informed consent using the study-specific Informed Consent Form
- Inclusion/Exclusion screening
- Document medical history and demographics
- Complete physical exam
- Complete vital signs (heart rate, blood pressure) and measure height and weight
- Medication assessment
- 12 lead electrocardiogram
  - Blood assays for full blood count, renal function, cardiac enzymes, creatine kinease (CK) & creatine kinease myocardial band (CK-MB) and/or Troponin (within 7 days of index procedure)

## **Index Procedure (PCI)**

Participants who meet the eligibility criteria will be randomized within 24 hours prior to their PCI.

### Peri-Procedure Study Medication Regimen

Follow standard institutional and/or stent manufacturer’s guidelines for recommended anticoagulation regimen.

### Data to be collected prior to initiating PCI

- Operator and assistant details
- Arterial access site and side
- Procedure start time (Sheath insertion)
- PCI Start Time (Guide Catheter Insertion)
- American Heart Association/American College of Cardiology lesion classification

### Data to be collected during the procedure

- Guidewire activation to guidewire retraction
- Fluoroscopy time
- Participant and staff radiation exposure (dose area product (DAP) and air kerma (AK))
- Lesion characteristics (eg tortuosity, calcification and bifurcation)
- Operator assessment of stent length
- Robotic assessment of stent length
- Contrast volume
- Procedure end time (Guide Catheter out)
- Equipment utilisation
- Pre and post PCI stenosis percentage
- Need for manual input (if required) and reason for this
- Number of times stent visualisation technique (eg stent boost or clearstent) used
- Use of robotic algorithms (techIQ) to assist lesion navigation
- Any adverse events
- Use of intracoronary physiology and/or intracoronary imaging
- Operator survey of task load

### Standard PCI Technique

PCI will be performed as per the Institutional PCI procedure. Operators will be encouraged to raise the operating table as high as possible and use shuttering and collimation techniques to limit X ray exposures to the patient and staff.

### CorPath Use Technique

The operator should refer to the CorPath GRX System Operator’s Manual for a detailed description of the system and relevant techniques.

To obtain proper data for analysis, the following procedure, with associated data collection milestones, is recommended, but in no case, dictates use of the CorPath GRX System in a manner different from that presented in the Operator’s Manual:

1. Obtain access using conventional percutaneous catheterization techniques.
2. Once arterial access is obtained, insert a standard guide catheter using conventional techniques. The guide catheter and guidewire of the physician’s choosing shall be used.
3. Manually advance and engage the guide catheter in the coronary artery. Perform angiography to assess the target lesion(s). Record target lesion characteristics such as percent diameter stenosis, etc.
4. Introduce and advance coronary guidewire utilizing CorPath GRX System to target lesion.
5. Record visual estimate of lesion segment (prior to pre-dilatation).
6. Introduce and advance the therapeutic coronary device (balloon/stent) using the CorPath GRX System. The delivery of interventional dilatation and stenting devices to the target lesion (pre-dilation, stent, post dilatation) will be done using the CorPath GRX System. Active guide management may be used to fine tune the placement of or reengage the guide catheter in the ostium of the coronary artery.
7. Measure target lesion using the CorPath GRX System as described in Section 3.5.
8. After stent placement, measure stent length using the CorPath GRX System as described in Section 3.5.
9. Retract balloon/stent system using CorPath GRX System.
10. Retract the guidewire using the CorPath GRX System.
11. Perform completion angiography to assess the target lesion final diameter stenosis and the condition of associated vasculature.
12. Record final fluoroscopy time.
13. Record time of guide catheter removal from the coronary vessel (procedure end time).

### Conversion to Manual Procedure Data Collection

The Investigator shall determine if it is necessary to convert to standard manual percutaneous techniques to complete the PCI. The Investigator should make this decision based on his/her medical assessment of the situation in accordance with the best interest of the participant. It is recommended that conversion to manual operation be performed at the discretion of the Investigator or if any of the following occur:

- The inability to navigate the guidewire or balloon/stent catheters using the CorPath GRX System as intended.
- Any clinical condition that requires rapid medical intervention.

The reason for conversion to manual should be reported by the operator and documented.

### Partial Robotic Assistance Data Collection

All planned and unplanned partial robotic assistance, e.g. rotational atherectomy, IVUS, etc., will be documented. The investigator will indicate whether the manual part of the procedure was planned, e.g. lesion prep done with OTW system or unplanned, e.g. stent was undeliverable due to severe calcification requiring rotational atherectomy.

### Post-Procedure Data Collection

**Acute Post Procedure Medication Regimen**

- Follow standard operator and institutional routines for recommended anticoagulation and anti-platelet regimens.

**Post Procedure Participant Evaluations**

- Clinical assessment and physical examination
- Record of all adverse events as described in Section 7.7
- If clinically indicated as determined by the investigator
  - 12-lead ECG
  - CK, CK-MB and/or Troponin

## **Day 3 or Discharge**

The following evaluations are to be performed post the Index procedure on Day 3 or discharge (whichever is sooner):

- Complete physical exam
- Complete vital signs (heart rate, blood pressure)
- Medication assessment
- 12 lead electrocardiogram
  - Blood assays for full blood count, renal function, cardiac enzymes, creatine kinease (CK) & creatine kinease myocardial band (CK-MB) and/or Troponin
- Adverse event assessment as described in Section 7.6
- Event assessment

## **Day only PCI patients**

Some patients will be suitable for same day discharge as their PCI procedure. In this instance, they will be asked to return the next day to the cathether laboratory clinic for a medical review as detailed in Section 7.3.

## **Day 30**

The following evaluations are to be performed post the Index procedure on Day 30 ± 5 days:

- Medication assessment
- Adverse event assessment as described in Section 7.6
- Event assessment

## **Day 365 or End of Study (EOS)**

The following evaluations are to be performed post the Index procedure on Day 365 ± 14 days or EOS:

- Medication assessment
- Adverse event assessment
- Event assessment

## **Adverse Events, Event Assessment and Serious Adverse Device Events**

### Adverse Events (AEs)

Study staff will closely monitor each participant for the development of device and/or procedure related adverse events. Adverse events that meet this criteria will continue to be monitored until day 30 or until resolution or the investigator deems them stable and unresolved.

Adverse Events of Special Interest (AESI) are those defined as MACE including cardiac death, clinically relevant MI after coronary revascularization (Q-wave or non-Q-wave myocardial infarction), or clinically driven target vessel revascularization (TVR).

### Event Assessments

This study is not using any unregistered products therefore only serious adverse events that meet the MACE definition will be reported from visit 2 until the end of study visit (visit 5). Event Assessments will be reviewed by the End-Point Adjudication Committee.

**Major Adverse Cardiac Event (MACE):** MACE is defined as cardiac death, Q-wave or non Q-wave myocardial infarction (MI), or clinically driven target vessel revascularization (TVR) by percutaneous or surgical methods.

**Cardiac Death:** Death due to cardiac causes. If the cause of death cannot be determined, it will be categorized as cardiac.

Myocardial Infarction (Protocol Definition):

Clinically relevant MI after coronary revascularization will be defined by the 4^th^ Universal Definition of PCI related MI. Events as defined by the Society for Cardiovascular Angiography and Interventions definition for periprocedural MI will also be recorded.

The 4th Universal Definition of PCI related MI defines:

- Participants with normal baseline cardiac biomarkers
  - Cardiac troponin (cTN) >5x the 99^th^ percentile of upper reference limit (URL)
- Participants with elevated baseline cardiac biomarkers
  - Rise in post procedure cTN by >20% and be at least >5x 99^th^ percentile of URL AND
  - At least one of the following
    - New ischaemic ECG changes
    - Development of new pathological Q waves
    - Imaging evidence of new loss of viable myocardium or new regional wall motion abnormality in a pattern consistent with an ischaemic aetiology
    - Angiographic findings consistent with a procedural flow limiting complication such as coronary dissection, occlusion of a major epicardial artery or a side branch occlusion/thrombus, disruption or collateral flow or distal embolisation

The Society for Cardiovascular Angiography and Interventions definition for periprocedural MI defines:

- Participants with normal baseline cardiac biomarkers
  - CK-MB >10x upper limit normal (ULN) or cTn (I or T) >70x ULN, or
  - CK-MB >5x ULN or cTn >35x ULN plus new Q-waves in >2 contiguous leads or LBBB
- Participants with elevated baseline cardiac biomarkers
  - Baseline biomarkers are stable or falling:
    - CK-MB >10x ULN or cTn (I or T) >70x ULN of **most recent pre-procedure level**, or
    - CK-MB >5x ULN or cTn >35x ULN of **most recent pre-procedure level** plus new Q-waves in >2 contiguous leads or LBBB
  - Baseline biomarkers have not been shown to be stable or falling: CK-MB (or cTn) rises by an absolute increment equal to those recommended above plus new ST-segment elevation or depression plus signs consistent with a clinically relevant MI, such as new onset or worsening heart failure or sustained hypotension.

**Target Vessel Revascularization (TVR):** Ischemia-driven repeat percutaneous intervention of the target vessel or bypass surgery of the target vessel. A TVR will be considered as ischemia-driven if the target vessel diameter stenosis is
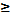
≥50% and any of the following are present:

- A positive functional study corresponding to the area served by the target vessel.
- Ischemic ECG changes at rest in a distribution consistent with the target vessel.
- Ischemic symptoms referable to the target lesion.

**In-Hospital MACE:** MACE that occurs within 72 hours of the procedure or prior to hospital discharge, whichever occurs first, in a participant treated with the CorPath GRX System.

### Serious Adverse Device Effect (SADE)

Serious adverse device effect (SADE) is any adverse event that meets the serious criteria as per the TGA [Therapeutic Goods (Medical Devices) Regulations 2002](https://www.legislation.gov.au/Series/F2002B00237) section 5.7

Any of the following device related adverse events will therefore be reported to the manufacturer:

- death of a patient, health care provider, user or other person; or
- a serious injury or serious deterioration to a patient, health care provider, user or other person, including;
- a life-threatening illness or injury;
- permanent impairment of a body function;
- permanent damage to a body structure; or
- a condition necessitating medical or surgical intervention to prevent permanent impairment of a body function or permanent damage to a body structure.
- near adverse event, it is sufficient that:
- an event associated with the device occurred; and
- if the event occurred again, it might lead to death or serious injury as outlined above.

### Event Relationships

Investigators will report the relationship of each event to the study device or procedure.

| Procedure related | An adverse event which, in the judgment of the Investigator, results as a consequence of the procedure and is not specifically related to use of the device. |
| --- | --- |
| Device related | An adverse event which, in the judgment of the Investigator, results as a consequence of the use of the device and is not specifically related to the treatment procedure |
| Unrelated | The adverse event is clearly not related to the device or procedure - i.e. another cause of the event is most plausible;  and/or a clinically plausible temporal sequence is inconsistent with the onset of the event. |

### Event Expectedness

Adverse events must be assessed as to whether they were anticipated to occur or unanticipated, meaning not anticipated based on current knowledge or found in the protocol, investigator brochure, product insert, or label. Categories are:

| Unanticipated | Nature or severity of the event is not consistent with information about the condition under study or intervention in the protocol, consent form, instructions for use or investigator brochure. |
| --- | --- |
| Anticipated | Event is known to be associated with the intervention or condition under study. |

### Event Severity

Investigators will be asked to report the severity of the adverse event, using the following definitions:

| Mild | Awareness of signs for symptoms but easily tolerated; are of minor irritant; causing no loss of time from normal activities |
| --- | --- |
| Moderate | Discomfort intense enough to cause interference with usual activities |
| Severe | Incapacitating with inability to do work or usual activites |

### Device Deficiency

Any device deficiencies must be reported to the TGA in accordance with Medical device safety reporting.

## **Reporting Procedures**

### General Reporting Requirements (AEs and Device Deficiencies)

- The following criteria must also be adhered to by the Investigator in the electronic data capture (EDC) system:
- Completion of separate Serious/ Adverse Event forms to document each event
- Completion of separate Device Observation forms for each device observation/ deficiency
- The forms must be electronically signed by the Investigator
- Supply to the appropriate authority upon authority’s request, any additional information related to the safety reporting of a particular event

### Reporting Requirements of SADEs

This study does not require serious adverse event reporting as all products used are being used within indication of the listing on the Australia Registry of Therapeutic Goods (ARTG). However in accordance with TGA requirements an automatic condition of inclusion under 5.7 of the [Therapeutic Goods (Medical Devices) Regulations 2002](https://www.legislation.gov.au/Series/F2002B00237) sponsors of a medical device report adverse events or near adverse events to the TGA [Incident Reporting and Investigation Scheme (IRIS)](https://www.tga.gov.au/node/289286).

# Data Management, Statistical Analysis and Record Keeping

## **Statistical Methods**

This is a double-arm, open-label, randomized controlled trial of the CorPath GRX System designed to evaluate the safety and effectiveness of the clinical and technical performance of the CorPath GRX System in the delivery and manipulation of coronary guidewires and stent/balloon catheters, and remote manipulation of guide catheters for use in PCI in comparison to standard of care. The analysis of data collected as part of the RCT will include, but may not be limited to, the Objectives and Measures outlined in Section 3.0 of this protocol. Continuous variables will be summarized using the number of observations, mean, standard deviation, median, min and max values. Categorical variables will be summarized with the number and percent in each category out of the number observed. Two-sided 95% confidence intervals may be calculated and two-sided p-values < 0.05 will be considered statistically significant.

The outcome of patient radiation exposure will be analysed using variance (ANOVA). As each participant may have multiple lesions, the outcome of clinical success at day 3 will be analysed using a two level multilevel models. Logit link will be used with a binomial distribution and restricted maximum likelihood (REML) will be used for estimation. Predictor variables in the models will include treatment group.

Technical success will analysed with Fisher’s exact test, comparing the proportion of participants with technical success between the two groups. Logistic regression will be used to compare technical success accounting for lesion complexity. For all other secondary outcomes, continuous outcomes will be measured using independent t-test (or non-parametric alternative depending on distributional assumptions), and categorical outcomes analysed by Fisher’s exact test. For outcomes measured repeatedly over time, multilevel models with an appropriate link functions will be used for analysis.

All statistical analyses will be performed using SAS software (version 9.4) or other widely accepted statistical or graphical software.

A total sample of 300 patients (150 in each group) is sufficient to detect a 20% reduction in radiation exposure to the patient during PCI, assuming a mean radiation exposure of 1284 mGy with standard deviation 180 mGy in the control group, and a standard deviation of 142.83 in the intervention group (29) with greater than 99% statistical power, and two sided statistical significance of 5%.

## **Data Management**

The site will maintain appropriate medical and research records for this trial, in compliance with regulatory and institutional requirements for the protection of confidentiality of participants. Source data are all information, original records of clinical findings, observations, or other activities in a study necessary for the reconstruction and evaluation of the trial.

## **Study Data Requirements**

To ensure data quality and completeness, all required data will be recorded on standardized electronic case report forms (eCRFs). eCRFs must be completed for each participant and once complete signed by the Investigator. The Investigator, or their authorized designee, is responsible for recording all study data in the eCRFs.

## **Data Entry**

Authorized study personnel will enter data directly into the eCRF. During data entry, each field is automatically subjected to data type verification and range checking. If an error occurs, the system ensures that the error is addressed before moving to the next entry field.

## **Final Data Analyses**

All datasets for analyses will undergo a final data cleaning procedure unique to each exported dataset.

## **Data Retention**

Data will be stored, accessed, archived and destroyed as per NSW Health Policies and the NHMRC National Statement on Ethical Conduct in Human Research. See section 9 for data protection. Study documents should be retained for a minimum of 15 years after the completion of the study (or greater according to site specific requirements). No records will be destroyed without the written consent of the Sponsor.

# Monitoring

## **Protocol Training**

Training on study protocol requirements will be provided for the entire study team at the initiation of the study. It is ultimately the responsibility of the Investigator to ensure all clinical site personnel participating in this study are adequately trained.

To ensure uniform data collection and protocol compliance, the study initiation visits to review the clinical protocol, techniques for the identification of eligible participants, instructions on in-hospital data collection, methods for soliciting data from alternative sources, and schedule for follow-up with study site personnel.

## **Device Training**

The Robotic PCI will only be performed by qualified investigators familiar with the CorPath^®^ GRX System. Training on the CorPath^®^ GRX System and procedure will be performed and documented for the site Investigator who is responsible for using the device.

## **Confidentiality and Protection of Study Files**

All data collection and reporting will follow strict adherence to Australian Privacy Legislation and NSW Health Policies for participant confidentiality.

All participants medical and procedural information will be kept on a password protected computer on an NSW Health Departmental password accessed network drive within a locked office. Once enrolled in the trial, participants will be allocated a unique identifier number. All documents containing identifiable information will also be password protected. It is important for participants to be re-identifiable for the purpose of reviewing medical records and any documents containing this information will only be accessible by study team. The re-identifiable/coded (it is possible to use the code to re-identify you) information held by the study team however will not be destroyed.

Any breaches in confidentiality will be reported in accordance with these policies.

# Data Quality Assurance

## **Clinical Event Handling**

This RCT will collect data on participants undergoing standard treatments and procedures. Investigators will be asked to provide documentation of events on the eCRFs related to specific procedural events and device complications. The site will follow their routine hospital procedures for adverse event handling, as necessary.

## **Study Endpoint Event Adjudication**

An independent events committee will review all clinical events associated with the study endpoints. Adjudication will be based on narratives and source data supplied by the sites. Source data that may be collected for review include, but are not limited to, de-identified angiograms (diagnostic and procedural), catheterization lab report, procedure report, discharge summary, lab reports, and pre- and post-procedure ECGs. The site may be contacted for queries and additional support documentation.

# Administrative Responsibilities

This study will be conducted in accordance with Good Clinical Practice, all applicable regulations and the NHMRC National Statement of Ethical Conduct in Human Research.

## **Human Research Ethics Committee (HREC)**

The Clinical Protocol, PICF, and associated documents and any other relevant documentation shall be reviewed and approved by the HREC prior to participant enrolment. All proposed changes, including administrative changes, to the protocol, PICF, associated documents and other relevant documentation must be reviewed and approved by the HREC prior to implementation.

Investigators are responsible for obtaining and maintaining annual renewal of the study by their HREC.

## **Informed Consent Procedures**

Informed consent is a process that is initiated prior to the individual’s agreeing to participate in the study and continues throughout the individual’s study participation. Participant Information and Consent forms (PICF) must be approved by the HREC and Research Governance Office at each site. Participants will be asked to read and review the document. An interpreter will be provided as per NSW Health Policy for any participant who is not fluent in English. The investigator will explain the study to the participant and answer any questions that may arise. A verbal explanation will be provided in terms suited to the participant’s comprehension of the purposes, procedures, and potential risks of the study and of their rights as research participants. Participants will have the opportunity to carefully review the written consent form and ask questions prior to signing. The participants should have the opportunity to discuss the study with their family or surrogates or think about it prior to agreeing to participate.

The participant will sign the informed consent document prior to any procedures being done specifically for the study. Participants must be informed that participation is voluntary and that they may withdraw from the study at any time, without prejudice. A copy of the informed consent document will be given to the participants for their records. The informed consent process will be conducted and documented in the source document (including the date), and the form signed, before the participant undergoes any study-specific procedures. The rights and welfare of the participants will be protected by emphasizing to them that the quality of their medical care will not be adversely affected if they decline to participate in this study.

All participants will provide informed consent prior to the PCI procedure.

## **Confidentiality**

All data collected concerning participants or their participation in this study will be considered confidential. All information collected about participants for the study will have the participant's name and address removed so that they cannot be recognised by it. The information will instead be labelled with a code. The Investigator (or delegate) is responsible for keeping a code list which makes it possible to link a participants assigned code to their name, so that in case of an emergency they can be identified and contacted. The Investigator will keep this code list in a secure location to protect participant privacy. The code list will be kept for the period required by applicable regulations and guidelines (a minimum of 15 years) or as per the institutions procedures. Each investigator must assure that the privacy of the participants, including their personal identity and all personal medical information, will be maintained at all times. In eCRFs and other documents participants will be identified not by their names, but by an identification code e.g. identification number. Personal medical information may be reviewed for the purpose of verifying data recorded on the eCRF. This review may be conducted by the trial monitor, properly authorised persons on behalf of South Western Sydney Local Health District. Personal medical information will always be treated as confidential.

All data used in the analysis and reporting of this evaluation will be used in a manner without identifiable reference to the participant.

## **Study Registration and Publication Policy**

Registration of this study is on the Australian New Zealand Clinical Trials Registry.

Results of the study will not be actively disseminated to participants, however, once the study results are available and have been published, participants will be given the opportunity to receive them upon request via the investigator. The Investigator is responsible for sharing these results with the participant as agreed by the participant. In addition, a summary of the results will be available on http://www.anzctr.org.au/

# References

1. Kim, K.P., et al., Occupational radiation doses to operators performing cardiac catheterization procedures. Health Phys, 2008. 94(3): p. 211-27.
2. Klein, L.W., et al., Occupational health hazards in the interventional laboratory: time for a safer environment. Catheter Cardiovasc Interv, 2009. 73(3): p. 432-8.
3. Vano, E., et al., Lens injuries induced by occupational exposure in non-optimized interventional radiology laboratories. Br J Radiol, 1998. 71(847): p. 728-33.
4. Andersen, A., et al., Work-related cancer in the Nordic countries. Scand J Work Environ Health, 1999. 25 Suppl 2: p. 1-116.
5. Blettner, M., et al., Medical exposure to ionising radiation and the risk of brain tumours: Interphone study group, Germany. Eur J Cancer, 2007. 43(13): p. 1990-8.
6. Carozza, S.E., et al., Occupation and adult gliomas. Am J Epidemiol, 2000. 152(9): p. 838-46.
7. Finkelstein, M.M., Is brain cancer an occupational disease of cardiologists? Can J Cardiol, 1998. 14(11): p. 1385-8.
8. Hardell, L., et al., Ionizing radiation, cellular telephones and the risk for brain tumours. Eur J Cancer Prev, 2001. 10(6): p. 523-9.
9. Roguin, A., J. Goldstein, and O. Bar, Brain tumours among interventional cardiologists: a cause for alarm? Report of four new cases from two cities and a review of the literature. EuroIntervention, 2012. 7(9): p. 1081-6.
10. Venneri, L., et al., Cancer risk from professional exposure in staff working in cardiac catheterization laboratory: insights from the National Research Council's Biological Effects of Ionizing Radiation VII Report. Am Heart J, 2009. 157(1): p. 118-24.
11. Wenzl, T.B., Increased brain cancer risk in physicians with high radiation exposure. Radiology, 2005. 235(2): p. 709-10; author reply 710-1.
12. Yonehara, S., et al., Clinical and epidemiologic characteristics of first primary tumors of the central nervous system and related organs among atomic bomb survivors in Hiroshima and Nagasaki, 1958-1995. Cancer, 2004. 101(7): p. 1644-54.
13. Ciraj-Bjelac, O., et al., Risk for radiation-induced cataract for staff in interventional cardiology: is there reason for concern? Catheter Cardiovasc Interv, 2010. 76(6): p. 826-34.
14. Jacob, S., et al., Occupational cataracts and lens opacities in interventional cardiology (O'CLOC study): are X-Rays involved? Radiation-induced cataracts and lens opacities. BMC Public Health, 2010. 10: p. 537.
15. Vano, E., et al., Radiation cataract risk in interventional cardiology personnel. Radiat Res, 2010. 174(4): p. 490-5.
16. Matanoski, G.M., et al., The current mortality rates of radiologists and other physician specialists: specific causes of death. Am J Epidemiol, 1975. 101(3): p. 199-210.
17. Zakeri, F., T. Hirobe, and K. Akbari Noghabi, Biological effects of low-dose ionizing radiation exposure on interventional cardiologists. Occup Med (Lond), 2010. 60(6): p. 464-9.
18. Johnson, L.W., R.J. Moore, and S. Balter, Review of radiation safety in the cardiac catheterization laboratory. Cathet Cardiovasc Diagn, 1992. 25(3): p. 186-94.
19. Miller, D.L., et al., New recommendations for occupational radiation protection. J Am Coll Radiol, 2012. 9(5): p. 366-8.
20. Preston, D.L., et al., Solid cancer incidence in atomic bomb survivors: 1958-1998. Radiat Res, 2007. 168(1): p. 1-64.
21. Preston, D.L., et al., Tumors of the nervous system and pituitary gland associated with atomic bomb radiation exposure. J Natl Cancer Inst, 2002. 94(20): p. 1555-63.
22. Rehani, M.M., Training of interventional cardiologists in radiation protection--the IAEA's initiatives. Int J Cardiol, 2007. 114(2): p. 256-60.
23. Dehmer, G.J., Occupational hazards for interventional cardiologists. Catheter Cardiovasc Interv, 2006. 68(6): p. 974-6.
24. Goldstein, J.A., et al., Occupational hazards of interventional cardiologists: prevalence of orthopedic health problems in contemporary practice. Catheter Cardiovasc Interv, 2004. 63(4): p. 407-11.
25. Beyar, R., et al., Remote-control percutaneous coronary interventions: concept, validation, and first-in-humans pilot clinical trial. J Am Coll Cardiol, 2006. 47(2): p. 296-300.
26. MacHaalany, MD, Jimmy, Eltigani Abdelaal, MD, and Olivier F. Bertrand, MD, PhD. "Guide Catheter Selection For Transradial PCI". Cardiac Interventions Today 2013: 45-48. Print.
27. Weisz G, et al. Safety and feasibility of robotic percutaneous coronary intervention. J Am Col Cardiol 2013;61:1596-600
28. Campbell PT, et al. The impact of precise robotic lesion length measurement on stent length selection: ramifications for stent savings. Cardiovasc Revasc Medicine 2016;16:348-350
29. Patel TM., et al. Comparison of robotic percutaneous coronary intervention with traditional percutaneous coronary intervention. Circ: Cardiovasc Inter 2020;13:e008888
30. Hart SG, Staveland LE. Development of NASA-TLX (Task Load Index): Results of Empirical and Theoretical Research. In: Hancock PA, Meshkati N, editors. Advances in Psychology [Internet]. North-Holland; 1988 [cited 2023 May 16]. p. 139–83. (Human Mental Workload; vol. 52). Available from: https://www.sciencedirect.com/science/article/pii/S0166411508623869
31. Nasa-Task Load Index (NASA-TLX); 20 Years Later - Sandra G. Hart, 2006 [Internet]. [cited 2023 May 23]. Available from: https://journals.sagepub.com/doi/10.1177/154193120605000909
32. Grier RA. How High is High? A Meta-Analysis of NASA-TLX Global Workload Scores. Proc Hum Factors Ergon Soc Annu Meet. 2015 Sep 1;59(1):1727–31.

# APPENDIX A. DEFINITIONS

*MACE Definitions*

**Major Adverse Cardiac Event (MACE):** MACE is defined as cardiac death, Q-wave or non Q-wave myocardial infarction (MI), or clinically driven target vessel revascularization (TVR) by percutaneous or surgical methods.

**Cardiac Death:** Death due to cardiac causes. If the cause of death cannot be determined, it will be categorized as cardiac.

Myocardial Infarction (Protocol Definition):

Clinically relevant MI after coronary revascularization is defined as:

- Participants with normal baseline cardiac biomarkers
  - CK-MB >10x ULN or cTn (I or T) >70x ULN, or
  - CK-MB >5x ULN or cTn >35x ULN plus new Q-waves in >2 contiguous leads or LBBB
- Participants with elevated baseline cardiac biomarkers
  - Baseline biomarkers are stable or falling:
    - CK-MB >10x ULN or cTn (I or T) >70x ULN of **most recent pre-procedure level**, or
    - CK-MB >5x ULN or cTn >35x ULN of **most recent pre-procedure level** plus new Q-waves in >2 contiguous leads or LBBB
- Baseline biomarkers have not been shown to be stable or falling: CK-MB (or cTn) rises by an absolute increment equal to those recommended above plus new ST-segment elevation or depression plus signs consistent with a clinically relevant MI, such as new onset or worsening heart failure or sustained hypotension.

**Target Vessel Revascularization (TVR):** Ischemia-driven repeat percutaneous intervention of the target vessel or bypass surgery of the target vessel. A TVR will be considered as ischemia-driven if the target vessel diameter stenosis is
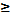
≥50% and any of the following are present:

- A positive functional study corresponding to the area served by the target vessel.
- Ischemic ECG changes at rest in a distribution consistent with the target vessel.
- Ischemic symptoms referable to the target lesion.

**In-Hospital MACE:** MACE that occurs within 72 hours of the procedure or prior to hospital discharge, whichever occurs first, in a participant treated with the CorPath GRX System.

*Other Definitions*

**Adverse Event Classification**

An adverse event is defined as any adverse change in health or undesirable clinical occurrence from the participant’s baseline whether it is considered device related or not.

An adverse event is considered serious if it results in death, is life-threatening, requires hospitalization or prolongation of existing hospitalization, results in persistent or significant disability/incapacity or is an important medical event which may jeopardize the participant and may require medical or surgical intervention to prevent one of the above outcomes.

**Procedure-Related Adverse Event**

An adverse event is procedure-related when, in the judgment of the Investigator, it is reasonable to believe that the event is not associated with the CorPath GRX System use. Instead, other products, surgical techniques, or medications required specifically for the procedure are deemed by Investigator as likely to have contributed to the occurrence of the event.

**Abrupt Closure:** The occurrence of new severely reduced flow (TIMI flow grade 0 or I) within the target vessel that persisted and required rescue by a non-assigned treatment strategy (including emergency surgery), or resulted in MI or death. Abrupt closure requires proven association with a mechanical dissection of the treatment site or instrumented vessel, coronary thrombus or severe spasm. Abrupt closure does not connote “no reflow” (due to microvasculature limitation), in which the epicardial artery is patent but had reduced flow. Abrupt closure also does not connote transient closure with reduced flow in which the index treatment application reversed the closure.

**Coronary Dissection** - Refers to a split or a tear in the wall of the [artery](http://www.wikidoc.org/index.php/Artery) which compresses or compromises the [lumen](http://www.wikidoc.org/index.php/Lumen) of the [artery](http://www.wikidoc.org/index.php/Artery) reducing blood flow.

**Coronary Guidewire Dissection** - A coronary dissection where a coronary guidewire is inadvertently positioned in a subintimal position or when stiff-tipped or hydrophilic-tipped guidewires are used to cross highly stenosed or totally occluded arteries.

**Coronary Perforation** - Coronary perforation occurs when a dissection or intimal tear propagates outward sufficient to completely penetrate the arterial wall. This may be caused by pre/post balloon dilatation or post stent deployment where the stent edge causes an edge perforation. Stent edge perforation will range in severity. Ellis classification of coronary perforation:

- Type I - Focal extraluminal crater without extravasation
- Type II - Pericardial or myocardial ‘blush’ without contrast agent
- Type III - Contrast agent ‘jetting’ through a frank (>1 mm) perforation

**Access Site Complication:** A complication occurring at the access site that requires either local intervention or blood transfusion.

**Subacute Thrombosis:** An abrupt closure of the target vessel that occurs after the index procedure is completed (and the participant has left the catheterization laboratory) and within 24 hours of the PCI.

**Threatened Abrupt Closure:** A grade B dissection and >50% diameter stenosis or any dissection of grade C or higher.

**Bleeding Requiring Transfusion:** Any blood loss requiring transfusion of blood products. Transfusion without clinical evidence of bleeding will be recorded separately.

**Coronary Artery Bypass Graft Surgery (CABG):** Classified as emergent, urgent, or elective as follows:

- **Elective**: the participant is clinically stable and the overall medical condition does not indicate the need for revascularization within forty-eight (48) hours.
- **Urgent**: the participant is clinically unstable and the condition warrants revascularization within two (2) to forty-eight (48) hours.
- **Emergent**: the participant is clinically unstable and the condition requires immediate revascularization within two (2) hours.

**Cardiogenic Shock:** Participant presents with systolic blood pressure <80 mm Hg for more than thirty (30) minutes unresponsive to fluids or requiring intravenous pressures or an intra-aortic balloon pump.

**CK:** Creatine kinase, or creatine phosphokinase.

**CK-MB:** An iso-enzyme of creatine phosphokinase (CK) with a distinct molecular structure specific as an indicator of myocardial cell injury. It is used to evaluate possible causes of chest pain, to detect and diagnose acute MI and re-infarction, and to monitor the severity of myocardial ischemia.

**Vascular Injuries:**

- **Dissection:** A tear in a vessel allowing blood to enter the wall of the vessel and split its layers. The result is either an intramural hematoma or aneurysmal dilatation.

Dissection classification per National Heart, Lung and Blood Institute:

- Type A: Radiolucent areas within the coronary lumen during contrast injection, with minimal or no persistence of contrast after dye has cleared.
- Type B: Parallel tracts or double lumen separated by a radiolucent area during contrast injection, with minimal or no persistence after dye has cleared.
- Type C: Contrast outside the coronary lumen, with persistence of contrast in the area after dye has cleared.
- Type D: Spiral luminal filling defects frequently with extensive contrast staining of the vessel.
- Type E: New persistent filling defects that may be caused by thrombus.
- Type F: Non type A-E dissection types that lead to impaired flow or total occlusion of the coronary artery.

**No Reflow:** Defined as a sustained or transient reduction in antegrade flow that is not associated with an obstructive lesion at the treatment site.

**Perforation:** Perforations are classified as follows:

- **Angiographic** **perforation**: Perforation detected by the clinical site at any point during the procedure.
- **Clinical** **perforation**: Perforation requires additional treatment beyond IV fluid administration (including efforts to seal the perforation or pericardial drainage), or resulting in significant pericardial effusion, abrupt closure, myocardial infarction, or death.

**Restenotic Lesion:** Defined as lesion in a vessel that had undergone a prior percutaneous treatment.

**Thrombus:** Discrete, mobile intraluminal filling defect with defined borders with or without staining.

Thrombosis: Academic research Consortium (ARC) definitions:

- **Definite stent thrombosis** requiring the presence of an acute coronary syndrome with angiographic or autopsy evidence of thrombus or occlusion**.**
- **Probable stent thrombosis** includes unexplained deaths within 30 days after the procedure or acute myocardial infarction involving the target-vessel territory without angiographic confirmation.
- **Possible stent thrombosis** includes all unexplained deaths occurring at least 30 days after the procedure.

**TIMI Flow:**

- **TIMI 0**: Dye fails to enter the microvasculature. There is either minimal or no ground glass appearance (“blush”) or opacification of the myocardium in the distribution of the culprit artery indicating lack of tissue level perfusion.
- **TIMI I**: Dye slowly enters but fails to exit the microvasculature. There is the ground glass appearance (“blush”) or opacification of the myocardium in the distribution of the culprit lesion that fails to clear from the microvasculature, and dye staining is present on the next injection (approximately thirty (30) seconds between injections).
- **TIMI** **II**: There is delayed entry and exit of dye from the microvasculature. There is the ground glass appearance (“blush”) or opacification of the myocardium in the distribution of the culprit lesion that is strongly persistent at the end of the washout phase (i.e. dye is strongly persistent after three (3) cardiac cycles of the washout phase and either does not or only minimally diminishes in intensity during washout).
- **TIMI** **III**: There is normal entry and exit of dye from the microvasculature. There is the ground glass appearance (“blush”) or opacification of the myocardium in the distribution of the culprit lesion that clears normally, and is either gone or only mildly/moderately persistent at the end of the washout phase (i.e. dye is gone or is mild/moderately persistent after three (3) cardiac cycles of the washout phase and noticeably diminishes in intensity during the washout phase), similar to that in an uninvolved artery. Blush that is of only mild intensity throughout the washout phase, but fades minimally is also classified as grade III.

**Total Occlusion:** True total occlusion is a lesion with TIMI 0 antegrade intraluminal flow and 100% diameter stenosis. A functional total occlusion is a lesion with TIMI I antegrade intraluminal flow and 99% diameter stenosis (functional TO) A chronic total occlusion is either of the above that has been present for at least 3 months.

**Troponin:** a complex of three regulatory proteins, measured levels of which can be used as a test of several different heart disorders, including [myocardial infarction](http://en.wikipedia.org/wiki/Myocardial_infarction).

**Unstable Angina:** Angina that increases in frequency, intensity, or duration, which occurs at rest, or which is new in onset.

**Vessel Characteristics:**

- **Angulation**: Vessel angle formed by the centerline through the lumen proximal to the stenosis and extending beyond it, and a second centerline in the straight portion of the artery distal to the stenosis measured in a non-foreshortened view.
- **Spasm**: Transient narrowing >50% diameter in a region where a <25% diameter stenosis had previously been.
- **Tortuosity**: Number of bends that must be traversed by a device to reach the target lesion.
- **Haziness**: Presence of radiolucencies within the arterial lumen not satisfying the criteria for thrombosis.

#### **Lesion Characteristics:**

- **Anastomotic**: Lesion located at the junction of a bypass graft and native vessel.
- **Aneurysm**: An expansion of the lumen in the region of maximum stenosis that extends with a wide or narrow mouth beyond the apparent normal contour.
- **Aorto-ostial**: Lesion which begins within 3-5 mm of the origin of a major epicardial artery. Ostial lesions represent a challenge to the interventional cardiologist because they often involve the wall of the aorta, they are often calcified, they may not fully dilate, and they are prone to restenosis.
- **Bifurcation**: Lesion located at the origin, immediately after, or branch that has a diameter ≥ 2mm.
- **Medina Classification**: Bifurcation (Medina) is classified when 50% lumen narrowing occurs within 3 mm of the bifurcation point.
- **Eccentricity**: A stenosis that has one of its luminal edges in the outer one-quarter of the apparent normal lumen.
- **Calcification**: Readily apparent densities noted within the apparent vascular wall at the site of the stenosis. Calcification is classified as none/mild, moderate when densities noted only during the cardiac cycle prior to contrast injection, and severe when densities noted without cardiac motion prior to contrast injection generally involving both sides of the arterial wall.
- **Intimal Flap**: Extrusion of tissue extending from the arterial surface into the lumen.
- **Irregularity**: Lesion borders with abnormal margins based on the presence of an ulceration, aneurysm, or intimal flap.
- **Length**: “Shoulder to shoulder” distance, which is measured from the proximal shoulder to the distal shoulder of a lesion in the projection that shows the most elongated view of the stenosis.
- **Discrete**: Lesion length <10.0 mm.
- **Tubular/Focal**: Lesion length >10.0 mm and <20.0 mm.
- **Diffuse**: Lesion length >20.0 mm.
- **Location**: Designated as ostial, proximal, mid and distal.
- **Ostial**: Lesions that begin within 3.0 mm of the origin of the artery.
- **Ulceration**: A small crater or flap in a lesion.

**ACC/AHA Lesion Characteristics (Type A, B and C):**

- *Type A Lesions (high success, >85%; low risk)*: Discrete (<10mm length), concentric, readily accessible, nonangulated segment ,45^o^, smooth contour, little or no calcification, less than totally occlusive, non-ostial in location, no major branch involvement and absence of thrombus.
- *Type B (B1 and B2) Lesions (moderate success, 60 - 85%; moderate risk)*: Tubular (10-20 mm length), eccentric, moderate tortuosity of proximal segment, moderately angulated, 45 - 90^o^ , irregular contour, moderate or heavy calcification, ostial in location, bifurcation lesions requiring double guidewires, some thrombus present and total occlusion < 3 months old. Type B2 lesion classification has more than one characteristic above.
- *Type C Lesions (low success, < 60%; high risk)*: Diffuse (>2 cm length), excessive tortuosity of proximal segment, extremely angulated, > 90^o^, inability to protect major side branch, degenerated vein grafts with friable lesions and total occlusions > 3 months old.
